# Impact of CT acquisition settings on the stability of radiomic features and the performance of pulmonary nodule classification models

**DOI:** 10.1186/s13244-025-02179-z

**Published:** 2026-01-05

**Authors:** Qian Zhou, Chengting Lin, Jinyi Jiang, Yuwei Li, Yue Yu, Shiyang Huang, Chaokang Han, Liting Shi, Lei Shi

**Affiliations:** 1 Postgraduate Training Base Alliance of Wenzhou Medical University (Zhejiang Cancer Hospital), Hangzhou, China; 2 Department of Radiology, Zhejiang Cancer Hospital, Hangzhou, China; 3 The Second School of Clinical Medicine, Zhejiang Chinese Medical University, Hangzhou, China

**Keywords:** CT reconstruction parameters, Image transmission, Model generalizability, Pulmonary nodule diagnosis, Radiomic feature stability

## Abstract

**Objectives:**

To evaluate the stability of radiomic features under different CT acquisition settings and investigate its impact on diagnostic model performance and generalizability.

**Materials and methods:**

198 patients with 1227 pulmonary nodules underwent chest CT scans using varied settings (three slice thicknesses, two reconstruction matrices, six convolution kernels, two transmission methods). 1394 radiomic features were extracted per nodule. Feature stability was evaluated using the Intraclass Correlation Coefficient (ICC, stable: ICC ≥ 0.8, intermediate stable: 0.4 < ICC < 0.8, unstable: ICC ≤ 0.4). Four diagnostic models (Full-feature, Stable, Unstable, Intermediate stable) were developed using two datasets (lung cancer screening, *n* = 184; clinical scenarios, *n* = 1192). In addition, three combination models were constructed for ablation analysis. Model performance and generalizability were assessed via fivefold cross-validation and independent test sets with different CT parameters.

**Results:**

Slice thickness and image transmission methods had the greatest and least impacts on feature stability (7.0% and 83.0% stable features, respectively). In training and validation sets, the Full-feature and Intermediate stable models showed higher AUCs than the Stable and Unstable models (*p* < 0.05). However, in test sets with varying CT parameters, the Stable model maintained consistent performance (AUC: 0.693–0.728), while the Unstable model exhibited the greatest variability (AUC: 0.523–0.800). Notably, the Full-feature and Intermediate stable models largely predicted nodules as benign, exhibiting limited ability to discriminate malignant cases.

**Conclusion:**

Radiomic feature stability is significantly affected by CT reconstruction parameters, especially slice thickness. Models based on stable features demonstrate better generalizability across varying CT settings, underscoring the importance of assessing feature stability in radiomic-based diagnostics.

**Critical relevance statement:**

Radiomic feature stability is significantly affected by CT acquisition parameters. Stable radiomic features enhance diagnostic model consistency and reliability across diverse CT settings. Therefore, feature stability analysis and selection of stable features are crucial to enhance model generalizability and stability.

**Key Points:**

- How do CT settings affect radiomic feature stability and model performance?
- Feature stability varies with CT parameters, but stable features enhance model generalizability.
- Stable feature models boost diagnostic reliability and clinical applicability.

**Graphical Abstract:**

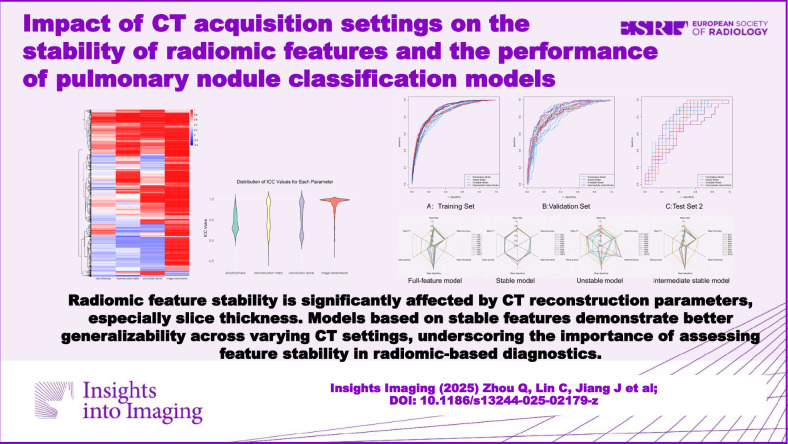

## Introduction

Lung cancer is the leading cause of cancer-related deaths worldwide [1]. Due to the lack of specific clinical symptoms in its early stages, most patients are diagnosed at an advanced stage, resulting in a global 5-year survival rate of only 10–20% [2, 3]. Therefore, early differentiation between benign and malignant pulmonary nodules is crucial for improving the prognosis of lung cancer patients [1]. Computed tomography (CT), currently the most common imaging modality [4, 5], plays an irreplaceable role in the screening and diagnosis of pulmonary nodules. In recent years, radiomics, which extracts quantitative features from medical images, has demonstrated significant value in early diagnosis, prognosis assessment, and treatment response prediction in oncology [6]. Multiple studies have confirmed that radiomic features derived from chest CT can provide valuable decision support for both the diagnosis and prognosis of patients with lung cancer or those undergoing lung cancer screening [4, 7–9].

Radiomic features are considered potential biomarkers in clinical decision support systems, and their stability is essential for ensuring their reliability. However, studies have shown that the radiomics research often lacks reproducibility, which may hinder its clinical applicability and generalizability [10]. The primary reason is that most radiomics studies rely on retrospectively collected data, where CT scanning protocols often lack standardization and may vary in terms of scanners, acquisition, and reconstruction parameters. Multiple studies have demonstrated that such variability in CT imaging parameters, including manufacturer, slice thickness, tube voltage, tube current, reconstruction kernel, reconstruction algorithm, and reconstruction matrix, significantly affects the stability of radiomic features [11–17]. Previous studies have primarily used phantoms to analyze the impact of CT parameter changes on radiomic feature stability [11, 13, 18–22]. While this approach allows for flexible adjustment of scanning parameters, phantoms do not fully represent the biological characteristics of real clinical cases. Moreover, existing research has mainly focused on radiomic feature stability; the impact of parameter-induced feature variability on model performance remains underexplored and warrants further investigation.

Therefore, this study aims to investigate how variations in CT acquisition settings affect the stability of radiomic features of pulmonary nodules in clinical routine CT scans acquired from the same patients. Based on this analysis, we constructed pulmonary nodule classification models using all, stable, unstable, or intermediately stable features. In addition, three combination models incorporating different feature categories were developed for an ablation study. These models were assessed to evaluate the impact of feature stability on model performance and generalizability across different CT acquisition settings.

## Materials and methods

### Study design and datasets

**Fig. 1:**
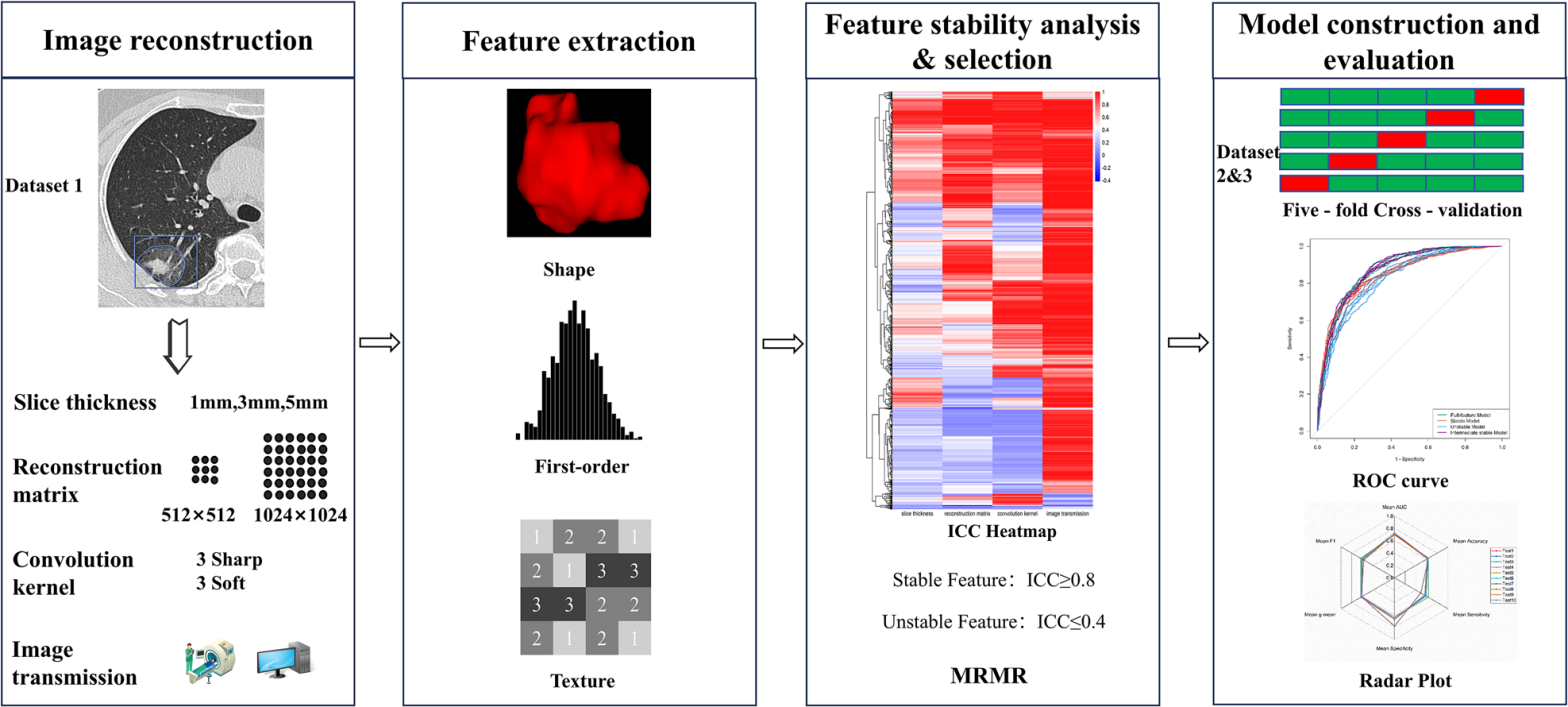
Overview of study design. ICC, intraclass correlation coefficient; ROC, receiver operating characteristic; MRMR, minimum redundancy maximum relevance

This study was approved by the Institutional Review Board of Zhejiang Cancer Hospital (IRB-2025-507). The overall design of this study is illustrated in Fig. 1. Three datasets were included in this study, as detailed below. They shared consistent criteria with respect to nodule size (5–30 mm) and image quality. Cases with poor image quality or substantial imaging artifacts were excluded.

### Dataset 1

Dataset 1 was collected from patients who underwent chest CT examinations using United Imaging equipment (May–December 2024), and for whom raw CT data were available. Exclusions: (1) history of other malignancies or prior lung surgery; (2) nodules that were not visible across all CT acquisition settings (Table 1). A total of 1227 pulmonary nodules from 198 patients were ultimately included. CT images were then reconstructed according to the study parameters (Table 1).

**Table 1:** Summary of CT reconstruction parameter variations in this study

Group	Slice thickness, mm	Reconstruction matrix	Convolution kernel	Image transmission
S1	1	1024 × 1024	B_SHARP_C	PACS
S2	1	1024 × 1024	B_SHARP_C	Scanner console
S3	3	1024 × 1024	B_SHARP_C	Scanner console
S4	5	1024 × 1024	B_SHARP_C	Scanner console
S5	1	1024 × 1024	B_SHARP_A	Scanner console
S6	1	1024 × 1024	B_VSHARP_D	Scanner console
S7	1	1024 × 1024	B_SOFT_C	Scanner console
S8	1	1024 × 1024	B_SOFT_F	Scanner console
S9	1	1024 × 1024	B_VSOFT_A	Scanner console
S10	1	512 × 512	B_SHARP_C	Scanner console

*PACS* Picture Archiving and Communication System

### Dataset 2

Dataset 2 included low-dose CT scans collected in a Wenling lung cancer screening program (2019–2020), with 3-year follow-up. The study included malignant cases diagnosed by surgical pathology within 1 year after the CT examinations. Benign cases, defined as individuals who remained free of lung cancer within 3 years after baseline screening, were matched to malignant cases at a 1:1 ratio using nearest-neighbor matching based on propensity scores estimated via logistic regression, with age and sex as matching variables. After rigorous selection, Dataset 2 included 184 patients with solitary nodules (92 benign, 92 malignant). If a patient had multiple nodules, only the largest one was selected for analysis.

### Dataset 3

Dataset 3 was derived from a surgical lung case database previously compiled by our research team. Inclusion criteria were: (1) pathological confirmation by surgical resection, and (2) a thin-slice chest CT (slice thickness ≤ 2 mm) performed within 1 month prior to surgery. Exclusion criteria included: (1) history of prior lung surgery, (2) any invasive procedure (e.g., biopsy, bronchoscopy) within 3 months prior to CT, and (3) for benign patients, a history of other malignancies. We selected a cohort of benign and malignant patients matched 1:1 by age and sex using nearest-neighbor matching based on propensity scores estimated via logistic regression. In total, Dataset 3 included 1192 patients with 1192 nodules.

### Image acquisition

**Fig. 2:**
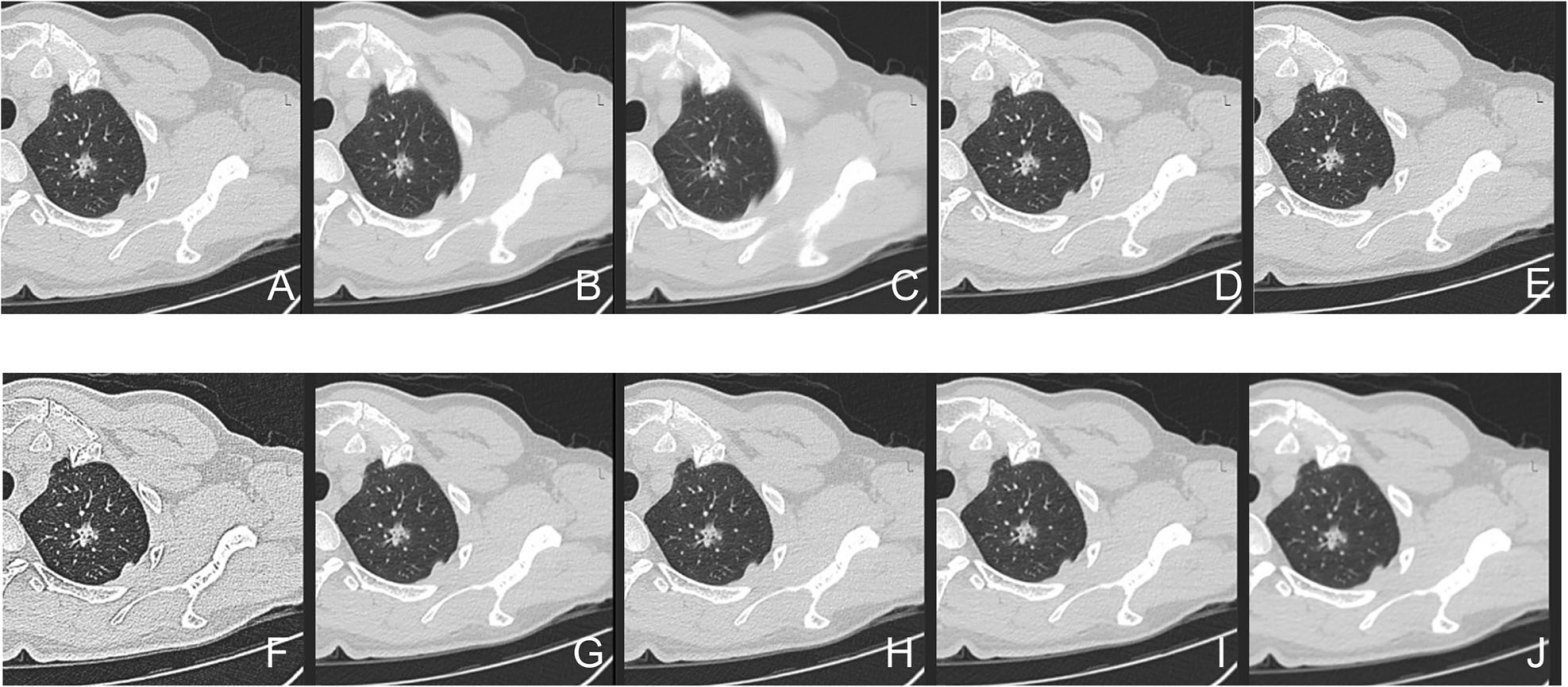
Images reconstructed using different parameters are shown. Except for F, which was exported from PACS, all others were exported directly from the workstation. **A** 1 mm, B_SHARP_C, 1024 × 1024; **B** 3 mm, B_SHARP_C, 1024 × 1024; **C** 5 m, B_SHARP_C, 1024 × 1024; **D** 1 mm, B_SHARP_C, 512 × 512; **E** 1 mm, B_SHARP_C, 1024 × 1024 (PACS); **F** 1 mm, B_VSHARP_D, 1024 × 1024; **G** 1 mm, B_SHARP_A, 1024 × 1024; **H** 1 mm, B_SOFT_F, 1024 × 1024; **I** 1 mm, B_SOFT_C, 1024 × 1024; **J** 1 mm, B_VSOFT_A, 1024 × 1024

Dataset 1 chest CTs were performed on a United Imaging Healthcare 760 scanner following institutional protocols (helical scan, automatic tube current modulation, 120 kV). Raw data were reconstructed into nine image sets (Table 1) varying in slice thickness, matrix, and kernel, with images exported directly from the CT console and picture archiving and communication system (PACS) (1 mm, 1024 × 1024, B_SHARP_C kernel) (Fig. 2). Dataset 2 low-dose CT scans used 120 kV, 40 mA, 1 mm slice thickness, 512 × 512 matrix, and CHEST_LUNG kernel. Dataset 3 was acquired from multiple CT devices (GE, Siemens, Philips, United Imaging), with 100–130 kV voltage, automatic tube current modulation, 1–2 mm slice thickness, and either 512 × 512 or 1024 × 1024 reconstruction matrix. All data were acquired from non-contrast-enhanced lung window images.

### Image segmentation and feature extraction

Pulmonary nodule segmentation in Dataset 1 and Dataset 2 was performed using a deep learning-based automated system on reconstructed lung window images [23]. Masks in Dataset 1 were generated on standard reconstructions (1 mm slice thickness, 1024 × 1024 matrix, B_SHARP_C convolution kernel, exported from the CT console) and resampled toother CT acquisition settings using nearest-neighbor interpolation, with the corresponding reconstructed images serving as reference (a representative segmentation example shown in Supplementary Fig. 1). Dataset 3 were transmitted to the Dr. Wise system, where nodules were semi-automatically detected and segmented at the lung window. The initial masks were automatically generated after an experienced radiologist (8 years in chest CT) delineated the nodule region and were then reviewed slice-by-slice by the same radiologist if needed. Accurate segmentation required the boundary line to follow the nodule edge precisely, except for the top or bottom slices with very small areas, where minor inaccuracies were acceptable [24, 25].

In this study, radiomic features of pulmonary nodules were extracted using version 3.1.0 of the Pyradiomics package, yielding a total of 1394 features for each nodule. The feature set included 106 original features (14 shape features, 18 first-order features, and 74 texture features), 736 wavelet features, 276 Laplacian of Gaussian (Log) features (with sigma values of 1.0, 2.0, and 3.0), 92 square features, 92 square-root features, and 92 exponential features. Prior to feature extraction, image intensities were scaled to a range of 0 to 2048, with a bin width of 25. The image was resampled to an isotropic voxel spacing of 1 × 1 × 1 mm using B-spline interpolation.

### Feature stability analysis

Dataset 1 was used for radiomic feature stability analysis. The intraclass correlation coefficient (ICC) [26, 27] was used to assess feature stability, based on a single-rating [k = 1], absolute-agreement, 2-way mixed-effects model. In line with prior studies [28–30], features with ICC ≥ 0.8 were considered stable, while those with ICC ≤ 0.4 were considered unstable, with 0.4 < ICC < 0.8 were considered intermediate stable. All statistical analyses were performed using R software (version 4.3.1).

### Model development and validation

Datasets 2 and 3, representing lung cancer screening and clinical scenarios, respectively, were combined to construct the training-validation cohort for model development. An independent test cohort was constructed using 48 surgically confirmed pulmonary nodules (benign: malignant = 24:24) from Dataset 1, with cases matched 1:1 by age and sex. To assess model generalizability under varying CT acquisition parameters, this cohort was further divided into ten test subsets based on variations in slice thickness, reconstruction matrix, convolution kernel and transmission methods. S1–S10 groups in Table 1 correspond to Test Sets 1–10, respectively, with Test Set 2 serving as the reference dataset.

To ensure robust model development and performance estimation, a fivefold cross-validation framework was adopted. In each fold, Datasets 2 and 3 were jointly split into training and validation sets. The corresponding Test Sets 1–10 from Dataset 1 were used for external testing. To ensure consistency, Z-score normalization was performed using parameters derived from the training data in each fold and applied to both the validation and external test data.

To investigate the impact of feature stability on model performance, seven pulmonary nodule classification models were constructed: a Full-feature model (all features), a Stable model (ICC ≥ 0.8), an Unstable model (ICC ≤ 0.4), and an Intermediate stable model (0.4 < ICC < 0.8). In addition, three combination models were developed as part of an ablation study—SI (stable + intermediate), SU (stable + unstable), and UI (intermediate + unstable). Next, the Minimum Redundancy Maximum Relevance (MRMR) algorithm was applied to select the top 20 ranked features for subsequent model development. The model was trained with Firth’s penalized log-likelihood loss and the iteratively reweighted least squares optimizer, with a maximum of 100 epochs for convergence.

### Model evaluation

A threshold of 0.5 was applied to the model outputs to classify predictions as positive or negative. Model performance was evaluated using multiple metrics, including area under the curve (AUC), accuracy, sensitivity, specificity, F1-score and the geometric mean (G-mean = $\surd {sensitivity}\times {specificity}$). Model performance was evaluated from two complementary perspectives: First, comparative performance analysis was conducted by evaluating each model on the training set, validation set, and Test Set 2 in each fold of fivefold cross-validation. Second, generalizability analysis was performed by applying the trained models from each fold to Test Sets 1–10. For statistical analysis, pairwise comparisons of AUCs were performed using the DeLong test, while sensitivity and specificity differences between models were evaluated using the McNemar test. A *p*-value < 0.05 was considered statistically significant.

## Results

### Feature stability analysis

Dataset 1 included 198 patients (mean age ± standard deviation: 60.33 ± 12.57 years) with a total of 1227 pulmonary nodules. The average nodule diameter was 9.31 ± 4.90 mm (Table 2), as measured by the original_shape_Maximum3DDiameter feature.

**Table 2:** Patient characteristics

	Dataset 1	Dataset 2	Dataset 3	Test set
	Patients, nodules (198, 1227)	Patients, nodules (184, 184)	Patients, nodules (1192, 1192)	Patients, nodules (46, 48)
Age, years (mean ± SD)	60.33 ± 12.57	61.27 ± 6.21	54.83 ± 10.26	56.87 ± 12.97
Sex				
Female	106 (53.5%)	86 (46.7%)	586 (49.2%)	26 (56.5%)
Male	92 (47.2%)	98 (53.3%)	606 (50.8%)	20 (43.5%)
Maximum diameter (mm)				
5 ≤ d ≤ 10	887 (78.7%)	52 (28.3%)	46 (3.9%)	5 (10.4%)
10 < d ≤ 20	265 (23.5%)	84 (45.7%)	692 (58.1%)	30 (62.5%)
20 < d ≤ 30	75 (6.7%)	48 (26.1%)	454 (38.1%)	13 (27.1%)
Benign	\	92 (50.0%)	596 (50.0%)	24 (50.0%)
Malignant	\	92 (50.0%)	596 (50.0%)	24 (50.0%)

In Dataset 1 and the Test set, one patient may have multiple nodules. Age and sex are calculated per patient, while nodule characteristics are calculated per nodule

**Fig. 3:**
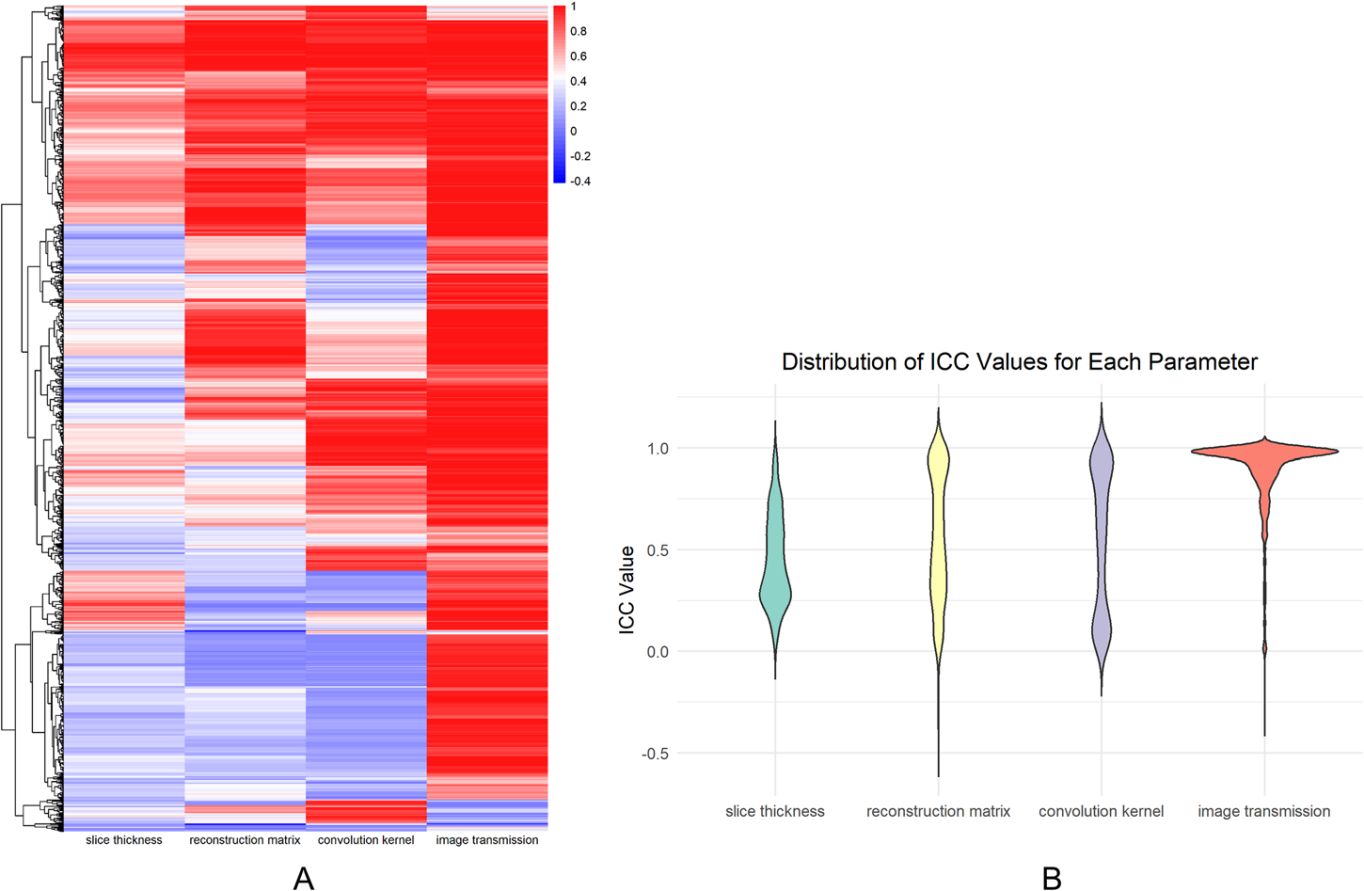
Heatmap and Violin plot. **A** Heatmap showing the distribution of radiomic feature values (columns) across different parameter variations (rows), with hierarchical clustering of features along the *y*-axis. **B** Violin plot of ICC values under different CT reconstruction parameters and data sources

Among these factors, slice thickness variation had the greatest impact on radiomic feature stability, while image transmission had minimal influence (Fig. 3). Varying slice thickness alone (1 mm, 3 mm, 5 mm) for the same patient’s CT images resulted in 7.0% of features (97 out of 1394) exhibiting ICC values ≥ 0.8. Changing only the reconstruction matrix (512 × 512 vs. 1024 × 1024) led to 31.8% of features (443 out of 1394) achieving ICC values ≥ 0.8. Variation in the convolution kernel (B_SHARP_A, B_SHARP_C, B_VSHARP_D, B_SOFT_C, B_SOFT_F, B_VSOFT_A) yielded 35.9% of features (501 out of 1394) with ICC values ≥ 0.8. When the image transmission method was altered (exported from the workstation vs. PACS), 83.0% of features (1157 out of 1394) demonstrated ICC values ≥ 0.8 (see Supplementary Table 1 for details). Notably, ICC values for wavelet features were consistently lower than those of other feature classes across different reconstruction settings (see Supplementary Table 2 for details).

**Fig. 4:**
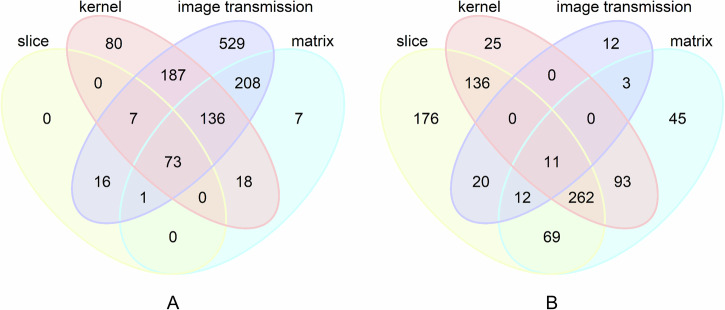
Venn diagrams illustrating the number of features (by region) among four sets. **A** ICC ≥ 0.8; **B** ICC ≤ 0.4

The overlap counts of features with ICC ≥ 0.8 and ICC ≤ 0.4 under all CT acquisition settings are illustrated in Fig. 4. Notably, only 5.2% of features (73 out of 1394) maintained stability (ICC ≥ 0.8). These stable features included 14 original features, 2 wavelet features, 48 log features, 4 square features, 4 square-root features, and 1 exponential feature (Supplementary Table 3). In contrast, only 0.8% of features (11 out of 1394) exhibited instability (ICC ≤ 0.4), all of which were wavelet features. (Supplementary Table 4).

### Performance comparison among four models

**Fig. 5:**
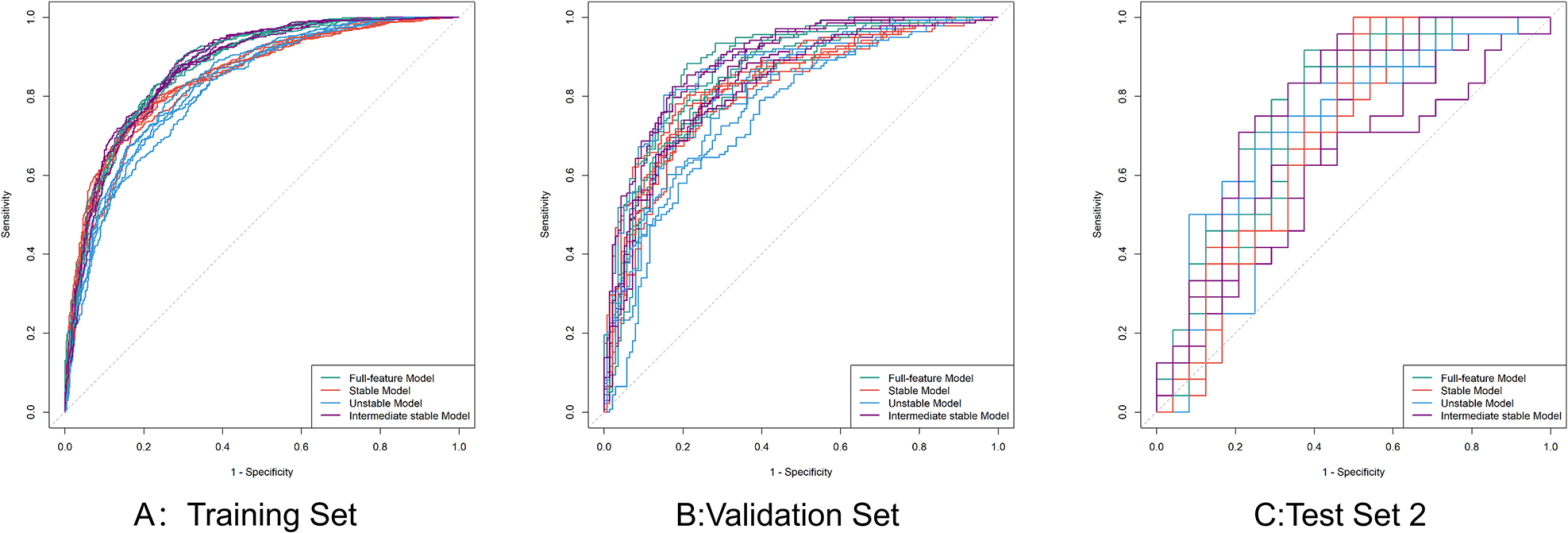
ROC curves of the ‘Full-feature model,’ ‘Stable model,’ and ‘Unstable model’ and ‘Intermediate stable model’ in the training set (**A**), validation set (**B**), and test set2 (**C**). Each model was trained using fivefold cross-validation, and each model produced five separate ROC curves corresponding to the five folds. In **A**–**C**, each line represents the performance of the model in one fold on the training set, validation set, and Test Set 2, respectively. ROC, receiver operating characteristic curve

Table 3 summarizes the average performance of the four models across different datasets. In the training and validation sets, the Full-feature model and the Intermediate stable model achieved significantly higher AUCs compared with the Stable model and the Unstable model (*p* < 0.05, Fig. 5). In fivefold cross-validation on the validation set, the specificity of the Full-feature, Intermediate stable, and Stable models was significantly higher than that of the Unstable model in most folds (≥ 3/5, *p* < 0.05). However, no significant differences were observed between the Full-feature and Intermediate stable models or between the Stable and Unstable models (see Supplementary Tables 5, 6 for details).

**Table 3:** Fivefold average performance metrics of the four models across each dataset

	Full-feature model	Stable model	Unstable model	Intermediate stable model
Train set				
AUC	0.872	0.845	0.827	0.873
Accuracy	0.788	0.771	0.744	0.785
Sensitivity	0.796	0.759	0.811	0.788
Specificity	0.780	0.783	0.676	0.782
G-mean	0.788	0.771	0.740	0.785
F1-score	0.790	0.768	0.760	0.786
Valid set				
AUC	0.858	0.828	0.808	0.856
Accuracy	0.775	0.766	0.739	0.775
Sensitivity	0.776	0.754	0.797	0.769
Specificity	0.773	0.778	0.680	0.780
G-mean	0.774	0.766	0.736	0.775
F1-score	0.775	0.763	0.753	0.773
Test set1				
AUC	0.752	0.728	0.701	0.703
Accuracy	0.538	0.633	0.650	0.538
Sensitivity	0.108	0.592	0.950	0.092
Specificity	0.967	0.675	0.350	0.983
G-mean	0.320	0.632	0.572	0.285
F1-score	0.188	0.617	0.731	0.158
Test set2				
AUC	0.737	0.703	0.725	0.694
Accuracy	0.538	0.621	0.608	0.538
Sensitivity	0.108	0.583	0.942	0.092
Specificity	0.967	0.658	0.275	0.983
G-mean	0.316	0.619	0.496	0.292
F1-score	0.186	0.605	0.706	0.162
Test set3				
AUC	0.694	0.708	0.700	0.640
Accuracy	0.529	0.612	0.625	0.513
Sensitivity	0.067	0.566	0.750	0.042
Specificity	0.992	0.658	0.500	0.983
G-mean	0.254	0.610	0.594	0.178
F1-score	0.123	0.594	0.666	0.096
Test set4				
AUC	0.647	0.700	0.608	0.578
Accuracy	0.517	0.633	0.546	0.529
Sensitivity	0.042	0.633	0.367	0.083
Specificity	0.992	0.633	0.725	0.975
G-mean	0.179	0.632	0.450	0.252
F1-score	0.097	0.633	0.399	0.182
Test set5				
AUC	0.719	0.696	0.545	0.634
Accuracy	0.504	0.625	0.529	0.500
Sensitivity	0.017	0.575	0.325	0
Specificity	0.992	0.675	0.733	1.000
G-mean	0.056	0.622	0.218	0
F1-score	0.148	0.604	0.453	NA
Test set6				
AUC	0.742	0.711	0.551	0.703
Accuracy	0.513	0.638	0.517	0.513
Sensitivity	0.034	0.492	0.034	0.050
Specificity	0.992	0.783	1.000	0.975
G-mean	0.163	0.619	0.164	0.190
F1-score	0.079	0.574	0.080	0.111
Test set7				
AUC	0.709	0.699	0.543	0.628
Accuracy	0.504	0.633	0.517	0.500
Sensitivity	0.017	0.592	0.292	0
Specificity	0.992	0.675	0.742	1.000
G-mean	0.056	0.632	0.156	0
F1-score	0.148	0.617	0.601	NA
Test set8				
AUC	0.746	0.697	0.715	0.743
Accuracy	0.533	0.625	0.612	0.529
Sensitivity	0.100	0.583	0.958	0.075
Specificity	0.967	0.669	0.267	0.983
G-mean	0.302	0.623	0.495	0.259
F1-score	0.173	0.608	0.712	0.132
Test set9				
AUC	0.715	0.702	0.523	0.566
Accuracy	0.500	0.612	0.525	0.500
Sensitivity	0.017	0.592	0.283	0
Specificity	0.983	0.633	0.767	1.000
G-mean	0.055	0.609	0.156	0
F1-score	0.143	0.602	0.595	NA
Test set10				
AUC	0.762	0.693	0.800	0.767
Accuracy	0.650	0.625	0.712	0.650
Sensitivity	0.483	0.591	0.642	0.475
Specificity	0.817	0.658	0.784	0.825
G-mean	0.624	0.624	0.709	0.623
F1-score	0.576	0.612	0.691	0.572

*AUC* area under the curve

In Test Set 2, no significant differences in AUC were detected among the four models (*p* > 0.05; mean AUCs of 0.737, 0.703, 0.725, and 0.694, respectively; see Supplementary Table 7). Notably, the Stable model demonstrated superior performance in accuracy and G-mean (accuracy: 0.621 vs. 0.538, 0.608, 0.538; G-mean: 0.619 vs. 0.316, 0.496, 0.292).

### Performance comparison across different CT parameter test sets

**Fig. 6:**
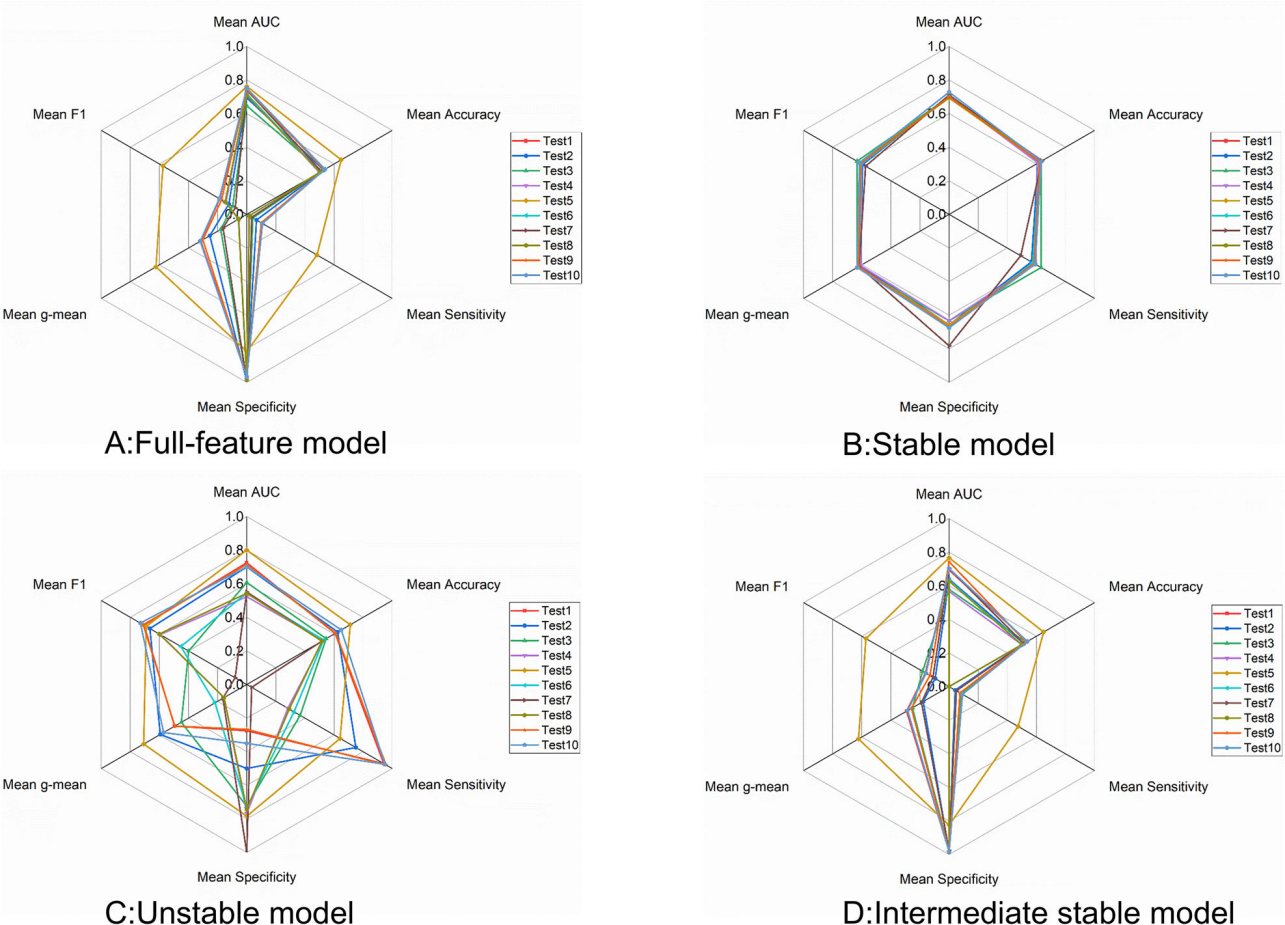
Radar plots showing the performance metrics of the Full-feature model (**A**), Stable model (**B**), Unstable model (**C**) and Intermediate stable model (**D**) across ten test sets (based on the mean of fivefold cross-validation)

In addition, across different CT reconstruction test sets (Supplementary Tables 8–11), the Stable model demonstrated consistent and robust performance (AUC: 0.693–0.728, Fig. 6), whereas the Unstable models showed the greatest variability (AUC: 0.523–0.800). Details are provided in Table 3.

The Full-feature and Intermediate stable models generally overpredicted benign cases, with Test Set 10 showing higher sensitivity than Test Set 2 (*p* < 0.05). The Unstable model varied considerably, showing significant differences in AUC, sensitivity, and specificity across multiple test sets (*p* < 0.05). In contrast, the Stable model maintained largely consistent performance, with only specificity in Test Set 9 differing significantly from Test Set 2.

In addition, an ablation study was conducted with three combination models (SI, SU, and UI); detailed results are presented in Supplementary Material, Section 2.

## Discussion

This study systematically analyzed the impact of CT reconstruction parameters and image transmission on the stability of radiomic features in pulmonary nodules from the same patients. Our results showed that both factors differently impacted feature stability, albeit to varying degrees. Moreover, the stability was clearly dependent on the feature category. To investigate whether feature stability influences the performance of malignancy classification models, we constructed multiple models and evaluated them across heterogeneous test sets. The Stable model consistently demonstrated more robust performance across datasets with varying CT parameters, whereas the other models exhibited greater variability. These findings indicated that radiomic feature stability is sensitive to differences in reconstruction. Prioritizing stable features during model development may enhance the generalizability and clinical utility of radiomics-based diagnostic models.

Among CT reconstruction parameters, slice thickness had the greatest impact on feature stability, with only 7.0% (97/1394) of features reaching ICC ≥ 0.8, consistent with previous studies [13, 14, 17, 31]. This may be attributed to partial volume effects and loss of image-plane information caused by larger slice thickness. Therefore, in radiomics studies, slice thickness should be carefully considered and kept as consistent as possible. In contrast, image transmission differences had minimal impact, with 83.0% (1157/1394) of features showing ICC ≥ 0.8, suggesting high information retention during image storage and transfer. Among feature types, Wavelet features were more susceptible to parameter changes and exhibited lower stability. Technically, wavelet transforms decompose the image into multiple frequency bands. High-frequency wavelet features are generally less stable than low-frequency features because they primarily capture fine textures and edge details, which are more susceptible to noise and variations in image spatial and density resolution, as also reported by Choe et al [32]. From a biological perspective, pulmonary nodules may also present heterogeneous microstructures that manifest differently across frequency scales, further contributing to variability.

In this study, Full-feature and Intermediate stable models performed similarly, as the Full-feature model largely overlapped with the Intermediate stable model (one stable, two unstable, and the rest intermediately stable features). The Stable model showed slightly lower performance in training and validation, likely due to excluding some informative but less stable features. However, the evaluation of model performance should place greater emphasis on generalizability rather than training accuracy. Across ten test sets with varying CT acquisition parameters, the Stable model consistently maintained robust performance (AUC: 0.693–0.728), whereas the Full-feature, Intermediate stable, and Unstable models exhibited considerable variability (AUC: 0.647–0.762, 0.566–0.767, and 0.523–0.800, respectively). Notably, the Unstable model demonstrated the poorest generalizability, which can be attributed to its reliance almost exclusively on wavelet features, which are sensitive to CT parameter variations and may contain redundancy and noise-related patterns.

While previously radiomics models have reported higher AUCs (0.800–0.930) under uniform acquisition conditions [33–35], their generalizability across heterogeneous imaging settings is often uncertain. In contrast, our Stable model maintained consistent performance across diverse CT acquisitions, which is clinically relevant since radiomics is often applied in multicenter or real-world environments. Although its AUC is modest compared with prior studies, it prioritizes robustness and reliability over potentially inflated results from a single dataset. These findings highlight that incorporating feature stability assessment—alongside feature selection and validation—can improve both generalizability and clinical applicability. The Stable model, by providing consistent predictions across heterogeneous CT settings, may serve as a reliable baseline tool for multicenter studies and clinical workflows where imaging protocols cannot always be standardized.

This study has several strengths. First, this study not only explored how CT reconstruction parameters affect the stability of radiomic features but also assessed the extent to which feature stability impacts the performance of radiomics-based models. Second, we developed malignancy classification models for pulmonary nodules in two clinical scenarios—screening settings and surgically confirmed cases—and further designed test datasets with varying CT reconstruction parameters to systematically evaluate the generalizability of these models under heterogeneous imaging conditions.

However, this study has some limitations. First, all data were acquired using a single CT scanner model. While this reduced hardware-related heterogeneity and enabled focus on acquisition parameters, it may limit the generalizability to data from other vendors. Second, the number of surgically confirmed benign nodules in the test set was relatively small. Moreover, the applicability of these models to other disease types requires further investigation. Future studies involving larger, multicenter datasets would be valuable for further validation of the findings.

In conclusion, CT reconstruction parameters significantly affect radiomic feature stability. Models based on stable features demonstrate better generalizability across varying CT settings. These results emphasize the importance of assessing feature stability in radiomic studies and suggest that prioritizing stable features in clinical practice may enhance the robustness and applicability of radiomics-based models.

## Supplementary information

ELECTRONIC SUPPLEMENTARY MATERIAL

## Data Availability

The datasets generated or analyzed during the study are not publicly available due to patient privacy concerns and institutional data protection policies.

## Abbreviations

AUC: Area under the curve
ICC: Intraclass correlation coefficient
PACS: Picture Archiving and Communication System
