# Supplementary material for: Impact of CT acquisition settings on the stability of radiomic features and the performance of pulmonary nodule classification models
Source: Insights Imaging. 2026 Jan 5;17:4. doi: 10.1186/s13244-025-02179-z (PMC12770142; doi:10.1186/s13244-025-02179-z)
Supplement: Supplementary file 1 — ELECTRONIC SUPPLEMENTARY MATERIAL [file 13244_2025_2179_MOESM1_ESM.pdf]

Impact of CT Acquisition Settings on the Stability of Radiomic Features and the Performance of Pulmonary Nodule Classification Models

ELECTRONIC SUPPLEMENTARY MATERIAL

Section 1. Supplementary Results for Main Text

Supplementary Table 1. Feature Type Frequency Across Different ICC Ranges Under Single-Parameter Variation.
Supplementary Table 2. ICC (Mean ± SD) of Radiomic Features Under Different Conditions.
Supplementary Table 3. Names of features with ICC ≥ 0.8 across all parameters.
Supplementary Table 4. Names of features with ICC ≤ 0.4 across all parameters.
Supplementary Table 5. Fold-wise DeLong and McNemar Test Results for Pairwise Comparisons among Four Models on the Training Set.
Supplementary Table 6. Fold-wise DeLong and McNemar Test Results for Pairwise Comparisons among Four Models on the Valid Set.
Supplementary Table 7. Fold-wise DeLong and McNemar Test Results for Pairwise Comparisons among Four Models on the Test Set2.
Supplementary Table 8. DeLong and McNemar Test Results for the Full-feature Model across 10 Test Sets (Reference: Test2).
Supplementary Table 9. DeLong and McNemar Test Results for the Stable Model across 10 Test Sets (Reference: Test2).
Supplementary Table 10. DeLong and McNemar Test Results for the Unstable Model across 10 Test Sets (Reference: Test2).
Supplementary Table 11. DeLong and McNemar Test Results for the Intermediate stable Model across 10 Test Sets (Reference: Test2).

Section 2. Additional Experiments

Supplementary Table 12. Five-fold average performance metrics of the additional three models across each dataset.
Supplementary Table 13. Fold-wise DeLong and McNemar Test Results for Pairwise Comparisons among three additional Models on the Training Set, Valid Set and Test Set2.
Supplementary Table 14. DeLong and McNemar Test Results for the SI Model across 10 Test Sets (Reference: Test2).
Supplementary Table 15. DeLong and McNemar Test Results for the SU Model across 10 Test Sets (Reference: Test2).
Supplementary Table 16. DeLong and McNemar Test Results for the UI Model across 10 Test Sets (Reference: Test2).

Supplementary Figures
Supplementary Figure 1. Segmentation results of images reconstructed using different CT parameters are presented. Except for F, which was exported from PACS, all others were exported directly from the workstation.A:1mm,B_SHARP_C,1024×1024;B:3mm,B_SHARP_C,1024×1024;C:5m,B_SHARP_C,1024×1024;D:1mm,B_SHARP_C,512×512;E:1mm,B_SHARP_C,1024×1024(PACS);F:1mm,B_VSHARP_D,1024×1024;G:1mm,B_SHARP_A,1024×1024;H:1mm,B_SOFT_F,1024×1024;I:1mm,B_SOFT_C,1024×1024;J:1mm,B_VSOFT_A,1024×1024.
Supplementary Figure 2. Radar plots showing the performance metrics of the SI model (A), SU model (B), and UI model (C) across ten test sets (based on the mean of five-fold cross-validation).

Section 1. Supplementary Results for Main Text

Supplementary Table 1. Feature Type Frequency Across Different ICC Ranges Under Single-Parameter Variation.

Group	Slice thickness			Reconstruction matrix			Convolution kernel			Image transmission		
	ICC ≥0.8	0.4 ＜ ICC ＜ 0.8	ICC ≤0.4	ICC ≥0.8	0.4 ＜ ICC ＜ 0.8	ICC ≤0.4	ICC ≥0.8	0.4 ＜ ICC ＜ 0.8	ICC ≤0.4	ICC≥ 0.8	0.4 ＜ ICC ＜ 0.8	ICC ≤0.4
All features (n=1394)	97	611	686	443	456	495	501	366	527	1157	179	58
Original(n=106)	14	53	39	58	38	10	40	46	20	88	18	0
Wavelet(n=736)	18	211	507	88	213	435	89	171	476	619	105	12
Log(n=276)	55	152	69	131	116	29	246	29	1	198	35	43
Square(n=92)	4	76	12	66	21	5	58	30	4	89	2	1
Squareroot(n=92)	4	46	42	44	41	7	22	47	23	73	19	0
Exponential(n=92)	2	73	17	56	27	9	46	43	3	90	0	2

Abbreviations: ICC, intraclass correlation coefficient

Supplementary Table 2. ICC (Mean ± SD) of Radiomic Features Under Different Conditions

Feature class	Slice thickness	Reconstruction matrix	Convolution kernel	Image transmission
Original	0.50±0.25	0.75±0.25	0.64±0.29	0.92±0.12
Wavelet	0.36±0.18	0.39±0.27	0.34±0.30	0.89±0.15
Log	0.57±0.23	0.73±0.25	0.91±0.10	0.79±0.30
Square	0.60±0.17	0.82±0.21	0.82±0.16	0.97±0.11
Squareroot	0.44±0.19	0.72±0.22	0.53±0.26	0.89±0.14
Exponential	0.59±0.18	0.75±0.21	0.79±0.17	0.97±0.12

Abbreviations: SD, Standard Deviation

Supplementary Table 3. Names of features with ICC ≥ 0.8 across all parameters.

original_shape_MajorAxisLength
original_shape_Maximum2DDiameterColumn
original_shape_Maximum2DDiameterRow
original_shape_Maximum2DDiameterSlice
original_shape_Maximum3DDiameter
original_shape_MeshVolume
original_shape_MinorAxisLength
original_shape_SurfaceArea
original_shape_VoxelVolume
original_firstorder_10Percentile
original_firstorder_Mean
original_firstorder_Median
original_firstorder_RootMeanSquared
original_firstorder_TotalEnergy
wavelet-LLL_firstorder_10Percentile
wavelet-LLL_firstorder_TotalEnergy
log-sigma-1-0-mm-3D_firstorder_Mean
log-sigma-1-0-mm-3D_firstorder_Median
log-sigma-1-0-mm-3D_firstorder_TotalEnergy
log-sigma-2-0-mm-3D_firstorder_10Percentile
log-sigma-2-0-mm-3D_firstorder_90Percentile
log-sigma-2-0-mm-3D_firstorder_InterquartileRange
log-sigma-2-0-mm-3D_firstorder_Maximum
log-sigma-2-0-mm-3D_firstorder_MeanAbsoluteDeviation
log-sigma-2-0-mm-3D_firstorder_Mean
log-sigma-2-0-mm-3D_firstorder_Median
log-sigma-2-0-mm-3D_firstorder_Minimum
log-sigma-2-0-mm-3D_firstorder_Range
log-sigma-2-0-mm-3D_firstorder_RobustMeanAbsoluteDeviation
log-sigma-2-0-mm-3D_firstorder_RootMeanSquared
log-sigma-2-0-mm-3D_firstorder_TotalEnergy
log-sigma-2-0-mm-3D_firstorder_Variance
log-sigma-2-0-mm-3D_glrlm_GrayLevelVariance
log-sigma-2-0-mm-3D_glszm_GrayLevelVariance
log-sigma-2-0-mm-3D_gldm_GrayLevelVariance
log-sigma-3-0-mm-3D_firstorder_10Percentile
log-sigma-3-0-mm-3D_firstorder_90Percentile
log-sigma-3-0-mm-3D_firstorder_Entropy
log-sigma-3-0-mm-3D_firstorder_InterquartileRange
log-sigma-3-0-mm-3D_firstorder_Maximum
log-sigma-3-0-mm-3D_firstorder_MeanAbsoluteDeviation
log-sigma-3-0-mm-3D_firstorder_Mean
log-sigma-3-0-mm-3D_firstorder_Median
log-sigma-3-0-mm-3D_firstorder_Minimum
log-sigma-3-0-mm-3D_firstorder_Range
log-sigma-3-0-mm-3D_firstorder_RobustMeanAbsoluteDeviation
log-sigma-3-0-mm-3D_firstorder_RootMeanSquared
log-sigma-3-0-mm-3D_firstorder_TotalEnergy
log-sigma-3-0-mm-3D_firstorder_Variance
log-sigma-3-0-mm-3D_glcm_Autocorrelation
log-sigma-3-0-mm-3D_glcm_ClusterTendency
log-sigma-3-0-mm-3D_glcm_JointAverage
log-sigma-3-0-mm-3D_glcm_SumSquares

log-sigma-3-0-mm-3D_glrlm_GrayLevelVariance
log-sigma-3-0-mm-3D_glrlm_HighGrayLevelRunEmphasis
log-sigma-3-0-mm-3D_glrlm_LongRunHighGrayLevelEmphasis
log-sigma-3-0-mm-3D_glrlm_RunEntropy
log-sigma-3-0-mm-3D_glrlm_ShortRunHighGrayLevelEmphasis
log-sigma-3-0-mm-3D_glszm_GrayLevelVariance
log-sigma-3-0-mm-3D_glszm_HighGrayLevelZoneEmphasis
log-sigma-3-0-mm-3D_glszm_SmallAreaHighGrayLevelEmphasis
log-sigma-3-0-mm-3D_gldm_GrayLevelVariance
log-sigma-3-0-mm-3D_gldm_HighGrayLevelEmphasis
log-sigma-3-0-mm-3D_gldm_SmallDependenceHighGrayLevelEmphasis
square_firstorder_Mean
square_firstorder_TotalEnergy
square_glszm_ZonePercentage
square_gldm_SmallDependenceEmphasis
squareroot_firstorder_10Percentile
squareroot_firstorder_Mean
squareroot_firstorder_Median
squareroot_firstorder_TotalEnergy
exponential_gldm_LowGrayLevelEmphasis

Abbreviations: ICC, intraclass correlation coefficient

Supplementary Table 4. Names of features with ICC ≤ 0.4 across all parameters.

wavelet-LLH_gldm_LargeDependenceLowGrayLevelEmphasis
wavelet-LHL_glcm_ClusterShade
wavelet-LHL_gldm_LargeDependenceLowGrayLevelEmphasis
wavelet-LHH_firstorder_Median
wavelet-LHH_glcm_ClusterShade
wavelet-HHL_firstorder_Mean
wavelet-HHL_firstorder_Median
wavelet-HHL_glcm_ClusterShade
wavelet-HHH_firstorder_Mean
wavelet-HHH_firstorder_Median
wavelet-HHH_glcm_ClusterShade

Abbreviations: ICC, intraclass correlation coefficient

Supplementary Table 5. Fold-wise DeLong and McNemar Test Results for Pairwise Comparisons among Four Models on the Training Set.

Fold	Full-AUC	Stable-AUC	P-value	95%CI
1	0.870	0.833	＜0.001*	[0.022 0.051]
2	0.877	0.852	＜0.001*	[0.010 0.040]
3	0.871	0.846	＜0.001*	[0.010 0.040]
4	0.863	0.844	0.018*	[0.003 0.034]
5	0.877	0.852	＜0.001*	[0.010 0.039]
Fold	Full-AUC	Unstable-AUC	P-value	95%CI
1	0.870	0.822	＜0.001*	[0.031 0.066]
2	0.877	0.836	＜0.001*	[0.024 0.059]
3	0.871	0.826	＜0.001*	[0.026 0.064]
4	0.863	0.814	＜0.001*	[0.030 0.067]
5	0.877	0.839	＜0.001*	[0.021 0.054]
Fold	Full-AUC	Intermediate-AUC	P-value	95%CI
1	0.870	0.877	0.010*	[-0.012 -0.002]
2	0.877	0.880	0.105	[-0.006 0.001]
3	0.871	0.868	0.438	[-0.004 0.010]
4	0.863	0.862	0.799	[-0.006 0.008]
5	0.877	0.877	0.943	[-0.007 0.007]
Fold	Stable-AUC	Unstable-AUC	P-value	95%CI
1	0.833	0.822	0.331	[-0.012 0.034]
2	0.852	0.836	0.118	[-0.004 0.037]
3	0.846	0.826	0.075	[-0.002 0.042]
4	0.844	0.814	0.007*	[0.008 0.052]
5	0.852	0.839	0.225	[-0.008 0.034]
Fold	Stable-AUC	Intermediate-AUC	P-value	95%CI
1	0.833	0.877	＜0.001*	[-0.059 -0.028]
2	0.852	0.880	＜0.001*	[-0.043 -0.013]
3	0.846	0.868	0.004*	[-0.037 -0.007]
4	0.844	0.862	0.024*	[-0.033 -0.002]
5	0.852	0.877	＜0.001*	[-0.039 -0.010]
Fold	Unstable-AUC	Intermediate-AUC	P-value	95%CI
1	0.822	0.877	＜0.001*	[-0.073 -0.037]
2	0.836	0.880	＜0.001*	[-0.062 -0.027]
3	0.826	0.868	＜0.001*	[-0.062 -0.023]
4	0.814	0.862	＜0.001*	[-0.066 -0.029]
5	0.839	0.877	＜0.001*	[-0.055 -0.020]

Fold	Full-Sensitivity	Stable-Sensitivity	P-value	Full-Specificity	Stable-Specificity	P-value
1	0.789	0.753	0.018*	0.775	0.764	0.550
2	0.800	0.769	0.047*	0.784	0.785	1.000
3	0.796	0.764	0.039*	0.782	0.785	0.910
4	0.788	0.755	0.045*	0.776	0.780	0.906
5	0.805	0.753	0.001*	0.784	0.802	0.289
Fold	Full-Sensitivity	Unstable-Sensitivity	P-value	Full-Specificity	Unstable-Specificity	P-value
1	0.789	0.824	0.073	0.775	0.673	＜0.001*
2	0.800	0.813	0.562	0.784	0.676	＜0.001*
3	0.796	0.798	1.000	0.782	0.665	＜0.001*
4	0.788	0.802	0.480	0.776	0.660	＜0.001*
5	0.805	0.818	0.505	0.784	0.704	＜0.001*
Fold	Full-Sensitivity	Intermediate-Sensitivity	P-value	Full-Specificity	Intermediate-Specificity	P-value
1	0.789	0.791	1.000	0.775	0.793	0.044*
2	0.800	0.795	0.579	0.784	0.776	0.502
3	0.796	0.771	0.045*	0.782	0.791	0.511
4	0.788	0.791	0.850	0.776	0.775	1.000
5	0.805	0.793	0.349	0.784	0.775	0.522
Fold	Stable-Sensitivity	Unstable-Sensitivity	P-value	Stable-Specificity	Unstable-Specificity	P-value
1	0.753	0.824	0.001*	0.764	0.673	＜0.001*
2	0.769	0.813	0.031*	0.785	0.676	＜0.001*
3	0.764	0.798	0.082	0.785	0.665	＜0.001*
4	0.755	0.802	0.014*	0.780	0.660	＜0.001*
5	0.753	0.818	0.001*	0.802	0.704	＜0.001*
Fold	Stable-Sensitivity	Intermediate-Sensitivity	P-value	Stable-Specificity	Intermediate-Specificity	P-value
1	0.753	0.791	0.019*	0.764	0.793	0.081
2	0.769	0.795	0.110	0.785	0.776	0.653
3	0.764	0.771	0.708	0.785	0.791	0.822
4	0.755	0.791	0.031*	0.780	0.775	0.815
5	0.753	0.793	0.010*	0.802	0.775	0.092
Fold	Unstable-Sensitivity	Intermediate-Sensitivity	P-value	Unstable-Specificity	Intermediate-Specificity	P-value
1	0.824	0.791	0.089	0.673	0.793	＜0.001*
2	0.813	0.795	0.373	0.676	0.776	＜0.001*
3	0.798	0.771	0.188	0.665	0.791	＜0.001*
4	0.802	0.791	0.614	0.660	0.775	＜0.001*
5	0.818	0.793	0.202	0.704	0.775	＜0.001*

* $P < 0.05$

Abbreviations: AUC, area under curve; CI, Confidence interval

Supplementary Table 6. Fold-wise DeLong and McNemar Test Results for Pairwise Comparisons among Four Models on the Vaid Set.

Fold	Full-AUC	Stable-AUC	P-value	95%CI
1	0.856	0.823	0.045*	[0.001 0.066]
2	0.835	0.805	0.049*	[0.000 0.060]
3	0.870	0.835	0.040*	[0.002 0.069]
4	0.894	0.854	0.014*	[0.008 0.072]
5	0.833	0.821	0.405	[-0.017 0.043]
Fold	Full-AUC	Unstable-AUC	P-value	95%CI
1	0.856	0.805	0.011*	[0.012 0.092]
2	0.835	0.765	0.004*	[0.022 0.117]
3	0.870	0.822	0.005*	[0.014 0.083]
4	0.894	0.871	0.165	[-0.009 0.056]
5	0.833	0.777	0.003*	[0.019 0.094]
Fold	Full-AUC	Intermediate-AUC	P-value	95%CI
1	0.856	0.854	0.652	[-0.009 0.014]
2	0.835	0.826	0.121	[-0.002 0.020]
3	0.870	0.878	0.303	[-0.023 0.007]
4	0.894	0.892	0.801	[-0.013 0.016]
5	0.833	0.831	0.742	[-0.014 0.020]
Fold	Stable-AUC	Unstable-AUC	P-value	95%CI
1	0.823	0.805	0.486	[-0.033 0.070]
2	0.805	0.765	0.148	[-0.014 0.093]
3	0.835	0.822	0.521	[-0.028 0.055]
4	0.854	0.871	0.407	[-0.056 0.023]
5	0.821	0.777	0.057	[-0.001 0.088]
Fold	Stable-AUC	Intermediate-AUC	P-value	95%CI
1	0.823	0.854	0.082	[-0.065 0.004]
2	0.805	0.826	0.159	[-0.051 0.008]
3	0.835	0.878	0.012*	[-0.076 -0.009]
4	0.854	0.892	0.014*	[-0.068 -0.008]
5	0.821	0.831	0.558	[-0.043 0.023]
Fold	Unstable-AUC	Intermediate-AUC	P-value	95%CI
1	0.805	0.854	0.020*	[-0.091 -0.008]
2	0.765	0.826	0.010*	[-0.107 -0.015]
3	0.822	0.878	0.001*	[-0.091 -0.022]
4	0.871	0.892	0.209	[-0.054 0.012]
5	0.777	0.831	0.014*	[-0.096 -0.011]

Fold	Full-Sensitivity	Stable-Sensitivity	P-value	Full-Specificity	Stable-Specificity	P-value
1	0.766	0.759	1.000	0.752	0.766	0.823
2	0.754	0.739	0.803	0.783	0.768	0.814
3	0.754	0.739	0.803	0.812	0.812	1.000
4	0.839	0.774	0.081	0.804	0.819	0.823
5	0.768	0.761	1.000	0.715	0.723	1.000
Fold	Full-Sensitivity	Unstable-Sensitivity	P-value	Full-Specificity	Unstable-Specificity	P-value
1	0.766	0.774	1.000	0.752	0.693	0.170
2	0.754	0.768	0.855	0.783	0.638	0.003*
3	0.754	0.783	0.522	0.812	0.725	0.025*
4	0.839	0.869	0.522	0.804	0.739	0.124
5	0.768	0.790	0.663	0.715	0.606	0.012*
Fold	Full-Sensitivity	Intermediate-Sensitivity	P-value	Full-Specificity	Intermediate-Specificity	P-value
1	0.766	0.752	0.617	0.752	0.766	0.617
2	0.754	0.754	1.000	0.783	0.761	0.248
3	0.754	0.775	0.546	0.812	0.826	0.617
4	0.839	0.825	0.773	0.804	0.797	1.000
5	0.768	0.739	0.386	0.715	0.752	0.302
Fold	Stable-Sensitivity	Unstable-Sensitivity	P-value	Stable-Specificity	Unstable-Specificity	P-value
1	0.759	0.774	0.860	0.766	0.693	0.165
2	0.739	0.768	0.584	0.768	0.638	0.009*
3	0.739	0.783	0.361	0.812	0.725	0.038*
4	0.774	0.869	0.021*	0.819	0.739	0.082
5	0.761	0.790	0.540	0.723	0.606	0.012*
Fold	Stable-Sensitivity	Intermediate-Sensitivity	P-value	Stable-Specificity	Intermediate-Specificity	P-value
1	0.759	0.752	1.000	0.766	0.766	1.000
2	0.739	0.754	0.803	0.768	0.761	1.000
3	0.739	0.775	0.302	0.812	0.826	0.789
4	0.774	0.825	0.121	0.819	0.797	0.646
5	0.761	0.739	0.628	0.723	0.752	0.522
Fold	Unstable-Sensitivity	Intermediate-Sensitivity	P-value	Unstable-Specificity	Intermediate-Specificity	P-value
1	0.774	0.752	0.719	0.693	0.766	0.078
2	0.768	0.754	0.850	0.638	0.761	0.009*
3	0.783	0.775	1.000	0.725	0.826	0.011*
4	0.869	0.825	0.307	0.739	0.797	0.186
5	0.79	0.739	0.281	0.606	0.752	0.002*

* $P < 0.05$

Abbreviations: AUC, area under curve; CI, Confidence interval

Supplementary Table 7. Fold-wise DeLong and McNemar Test Results for Pairwise Comparisons among Four Models on the Test Set2.

Fold	Full-AUC	Stable-AUC	P-value	95%CI
1	0.720	0.686	0.632	[-0.177 0.108]
2	0.705	0.686	0.742	[-0.133 0.094]
3	0.757	0.720	0.491	[-0.140 0.067]
4	0.714	0.707	0.912	[-0.130 0.116]
5	0.788	0.714	0.320	[-0.222 0.072]
Fold	Full-AUC	Unstable-AUC	P-value	95%CI
1	0.720	0.729	0.916	[-0.153 0.170]
2	0.705	0.670	0.700	[-0.211 0.142]
3	0.757	0.748	0.915	[-0.168 0.150]
4	0.714	0.747	0.664	[-0.116 0.182]
5	0.788	0.729	0.360	[-0.185 0.067]
Fold	Full-AUC	Intermediate-AUC	P-value	95%CI
1	0.720	0.708	0.626	[-0.061 0.037]
2	0.705	0.620	0.380	[-0.275 0.105]
3	0.757	0.714	0.236	[-0.115 0.028]
4	0.714	0.648	0.197	[-0.166 0.034]
5	0.788	0.781	0.748	[-0.049 0.035]
Fold	Stable-AUC	Unstable-AUC	P-value	95%CI
1	0.686	0.729	0.646	[-0.142 0.228]
2	0.686	0.670	0.865	[-0.196 0.165]
3	0.720	0.748	0.722	[-0.125 0.181]
4	0.707	0.747	0.654	[-0.135 0.215]
5	0.714	0.729	0.861	[-0.160 0.191]
Fold	Stable-AUC	Intermediate-AUC	P-value	95%CI
1	0.686	0.708	0.760	[-0.122 0.167]
2	0.686	0.620	0.555	[-0.285 0.153]
3	0.720	0.714	0.866	[-0.088 0.074]
4	0.707	0.648	0.464	[-0.217 0.099]
5	0.714	0.781	0.418	[-0.096 0.232]
Fold	Unstable-AUC	Intermediate-AUC	P-value	95%CI
1	0.729	0.708	0.820	[-0.158 0.200]
2	0.670	0.620	0.728	[-0.233 0.334]
3	0.748	0.714	0.662	[-0.121 0.190]
4	0.747	0.648	0.277	[-0.079 0.277]
5	0.729	0.781	0.448	[-0.186 0.082]

Fold	Full-Sensitivity	Stable-Sensitivity	P-value	Full-Specificity	Stable-Specificity	P-value
1	0.083	0.583	＜0.001*	1.000	0.708	0.016*
2	0.125	0.583	0.001*	0.917	0.625	0.016*
3	0.167	0.625	0.001*	0.958	0.667	0.016*
4	0.125	0.500	0.004*	0.958	0.667	0.016*
5	0.042	0.625	＜0.001*	1.000	0.625	0.004*
Fold	Full-Sensitivity	Unstable-Sensitivity	P-value	Full-Specificity	Unstable-Specificity	P-value
1	0.083	0.833	＜0.001*	1.000	0.458	＜0.001*
2	0.125	1.000	＜0.001*	0.917	0.125	＜0.001*
3	0.167	1.000	＜0.001*	0.958	0.292	＜0.001*
4	0.125	0.958	＜0.001*	0.958	0.250	＜0.001*
5	0.042	0.917	＜0.001*	1.000	0.250	＜0.001*
Fold	Full-Sensitivity	Intermediate-Sensitivity	P-value	Full-Specificity	Intermediate-Specificity	P-value
1	0.083	0.083	1.000	1.000	1.000	1.000
2	0.125	0.083	1.000	0.917	1.000	0.500
3	0.167	0.167	1.000	0.958	0.917	1.000
4	0.125	0.083	1.000	0.958	1.000	1.000
5	0.042	0.042	1.000	1.000	1.000	1.000
Fold	Stable-Sensitivity	Unstable-Sensitivity	P-value	Stable-Specificity	Unstable-Specificity	P-value
1	0.583	0.833	0.070	0.708	0.458	0.070
2	0.583	1.000	0.002*	0.625	0.125	＜0.001*
3	0.625	1.000	0.004*	0.667	0.292	0.004*
4	0.500	0.958	0.003*	0.667	0.250	0.002*
5	0.625	0.917	0.039*	0.625	0.250	0.004*
Fold	Stable-Sensitivity	Intermediate-Sensitivity	P-value	Stable-Specificity	Intermediate-Specificity	P-value
1	0.583	0.083	0.0004	0.708	1.000	0.016*
2	0.583	0.083	0.0004	0.625	1.000	0.004*
3	0.625	0.167	0.001	0.667	0.917	0.031*
4	0.500	0.083	0.002	0.667	1.000	0.008*
5	0.625	0.042	0.0001	0.625	1.000	0.004*
Fold	Unstable-Sensitivity	Intermediate-Sensitivity	P-value	Unstable-Specificity	Intermediate-Specificity	P-value
1	0.833	0.083	＜0.001*	0.458	1.000	＜0.001*
2	1.000	0.083	＜0.001*	0.125	1.000	＜0.001*
3	1.000	0.167	＜0.001*	0.292	0.917	＜0.001*
4	0.958	0.083	＜0.001*	0.250	1.000	＜0.001*
5	0.917	0.042	＜0.001*	0.250	1.000	＜0.001*

* $P < 0.05$

Abbreviations: AUC, area under curve; CI, Confidence interval

Supplementary Table 8. DeLong and McNemar Test Results for the Full-feature Model across 10 Test Sets (Reference: Test2).

**Test2-AUC**	**Test1-AUC**	P-value	95%CI
	0.738	0.336	[-0.053 0.018]
	0.715	0.444	[-0.037 0.016]
	0.781	0.210	[-0.062 0.014]
	0.757	0.077	[-0.092 0.005]
	0.769	0.359	[-0.022 0.060]
	**Test3-AUC**	P-value	95%CI
	0.684	0.552	[-0.084 0.157]
	0.655	0.390	[-0.064 0.165]
	0.734	0.613	[-0.065 0.110]
	0.686	0.588	[-0.073 0.128]
	0.712	0.331	[-0.078 0.230]
	**Test4-AUC**	P-value	95%CI
	0.627	0.263	[-0.070 0.258]
	0.648	0.399	[-0.076 0.191]
	0.750	0.898	[-0.100 0.114]
	0.641	0.298	[-0.064 0.210]
	0.569	0.039*	[0.011 0.427]
	**Test5-AUC**	P-value	95%CI
	0.691	0.402	[-0.039 0.099]
	0.688	0.589	[-0.046 0.080]
	0.747	0.704	[-0.043 0.064]
	0.700	0.718	[-0.061 0.089]
	0.771	0.509	[-0.034 0.069]
	**Test6-AUC**	P-value	95%CI
0.720	0.748	0.718	[-0.179 0.123]
0.705	0.736	0.644	[-0.164 0.101]
0.757	0.743	0.867	[-0.149 0.177]
0.714	0.714	1.000	[-0.178 0.178]
0.788	0.771	0.723	[-0.079 0.113]
	**Test7-AUC**	P-value	95%CI
	0.693	0.492	[-0.051 0.107]
	0.688	0.629	[-0.053 0.088]
	0.740	0.610	[-0.049 0.084]
	0.684	0.531	[-0.063 0.122]
	0.741	0.181	[-0.022 0.116]
	**Test8-AUC**	P-value	95%CI
	0.729	0.569	[-0.039 0.021]
	0.722	0.332	[-0.052 0.018]
	0.783	0.148	[-0.061 0.009]
	0.708	0.763	[-0.029 0.039]
	0.786	0.853	[-0.017 0.020]
	**Test9-AUC**	P-value	95%CI
	0.689	0.775	[ ]
	0.710	0.962	[ ]
	0.755	0.986	[ ]
	0.693	0.848	[ ]
	0.727	0.554	[ ]
	**Test10-AUC**	P-value	95%CI
	0.733	0.847	[-0.136 0.112]
	0.774	0.112	[-0.155 0.016]
	0.785	0.514	[-0.111 0.056]
	0.719	0.919	[-0.105 0.095]
	0.799	0.786	[-0.086 0.065]

Test2-Sensitivity	Test1-Sensitivity	P-value	Test2-Specitivity	Test1-Specitivity	P-value
	0.083	1.000		1.000	1.000
	0.083	1.000		0.917	1.000
	0.167	1.000		0.958	1.000
	0.125	1.000		0.958	1.000
	0.083	1.000		1.000	1.000
	**Test3-Sensitivity**	P-value		**Test3-Specitivity**	P-value
	0.042	1.000		1.000	1.000
	0.083	1.000		0.958	1.000
	0.083	0.500		1.000	1.000
	0.083	1.000		1.000	1.000
	0.042	1.000		1.000	1.000
	**Test4-Sensitivity**	P-value		**Test4-Specitivity**	P-value
	0.042	1.000		1.000	1.000
	0.083	1.000		0.958	1.000
	0.042	0.250		1.000	1.000
	0.042	0.500		1.000	1.000
	0	1.000		1.000	1.000
	**Test5-Sensitivity**	P-value		**Test5-Specitivity**	P-value
	0	0.500		1.000	1.000
	0.083	1.000		0.958	1.000
	0	0.125		1.000	1.000
	0	0.250		1.000	1.000
	0	1.000		1.000	1.000
	**Test6-Sensitivity**	P-value		**Test6-Specitivity**	P-value
0.083	0.042	1.000	1	1.000	1.000
0.125	0.042	0.500	0.917	0.958	1.000
0.167	0.042	0.250	0.958	1.000	1.000
0.125	0.042	0.500	0.958	1.000	1.000
0.042	0	1.000	1	1.000	1.000
	**Test7-Sensitivity**	P-value		**Test7-Specitivity**	P-value
	0	0.500		1.000	1.000
	0.083	1.000		0.958	1.000
	0	0.125		1.000	1.000
	0	0.250		1.000	1.000
	0	1.000		1.000	1.000
	**Test8-Sensitivity**	P-value		**Test8-Specitivity**	P-value
	0.083	1.000		1.000	1.000
	0.125	1.000		0.917	1.000
	0.167	1.000		0.958	1.000
	0.083	1.000		0.958	1.000
	0.042	1.000		1.000	1.000
	**Test9-Sensitivity**	P-value		**Test9-Specitivity**	P-value
	0	0.500		1.000	1.000
	0.083	1.000		0.917	1.000
	0	0.125		1.000	1.000
	0	0.250		1.000	1.000
	0	1.000		1.000	1.000
	**Test10-Sensitivity**	P-value		**Test10-Specitivity**	P-value
	0.542	0.001*		0.792	0.063
	0.458	0.008*		0.833	0.500
	0.542	0.004*		0.792	0.125
	0.542	0.002*		0.750	0.063
	0.333	0.016*		0.917	0.500

* $P < 0.05$

Abbreviations: AUC, area under curve; CI, Confidence interval

Supplementary Table 9. DeLong and McNemar Test Results for the Stable Model across 10 Test Sets (Reference: Test2).

**Test2-AUC**	**Test1-AUC**	P-value	95%CI
	0.720	0.129	[-0.080 0.010]
	0.712	0.283	[-0.074 0.022]
	0.740	0.385	[-0.062 0.024]
	0.729	0.368	[-0.072 0.027]
	0.741	0.183	[-0.069 0.013]
	**Test3-AUC**	P-value	95%CI
	0.705	0.487	[-0.073 0.035]
	0.701	0.493	[-0.060 0.029]
	0.717	0.848	[-0.032 0.039]
	0.707	1.000	[-0.038 0.038]
	0.708	0.781	[-0.031 0.042]
	**Test4-AUC**	P-value	95%CI
	0.677	0.841	[-0.076 0.093]
	0.696	0.711	[-0.065 0.045]
	0.715	0.867	[-0.056 0.066]
	0.700	0.844	[-0.062 0.076]
	0.714	1.000	[-0.059 0.059]
	**Test5-AUC**	P-value	95%CI
	0.684	0.826	[-0.014 0.017]
	0.684	0.841	[-0.015 0.019]
	0.710	0.221	[-0.006 0.027]
	0.703	0.641	[-0.011 0.018]
	0.700	0.115	[-0.003 0.031]
	**Test6-AUC**	P-value	95%CI
0.686	0.705	0.453	[-0.069 0.031]
0.686	0.688	0.948	[-0.054 0.050]
0.720	0.710	0.718	[-0.046 0.067]
0.707	0.733	0.451	[-0.094 0.042]
0.714	0.72	0.764	[-0.065 0.048]
	**Test7-AUC**	P-value	95%CI
	0.684	0.848	[-0.016 0.019]
	0.684	0.841	[-0.015 0.019]
	0.710	0.258	[-0.008 0.028]
	0.710	0.728	[-0.023 0.016]
	0.707	0.389	[-0.009 0.023]
	**Test8-AUC**	P-value	95%CI
	0.681	0.381	[-0.006 0.017]
	0.681	0.275	[-0.004 0.015]
	0.714	0.183	[-0.003 0.017]
	0.705	0.687	[-0.007 0.010]
	0.705	0.156	[-0.003 0.021]
	**Test9-AUC**	P-value	95%CI
	0.684	0.988	[ ]
	0.691	0.964	[ ]
	0.712	0.938	[ ]
	0.710	0.975	[ ]
	0.715	0.987	[ ]
	**Test10-AUC**	P-value	95%CI
	0.694	0.547	[-0.037 0.020]
	0.679	0.558	[-0.016 0.030]
	0.700	0.083	[-0.003 0.044]
	0.694	0.419	[-0.017 0.042]
	0.696	0.082	[-0.002 0.037]

Test2-Sensitivity	Test1-Sensitivity	P-value	Test2-Specitivity	Test1-Specitivity	P-value
	0.583	1.000		0.708	1.000
	0.583	1.000		0.667	1.000
	0.625	1.000		0.667	1.000
	0.542	1.000		0.667	1.000
	0.625	1.000		0.667	1.000
	**Test3-Sensitivity**	P-value		**Test3-Specitivity**	P-value
	0.583	1.000		0.708	1.000
	0.500	0.500		0.708	0.500
	0.583	1.000		0.625	1.000
	0.583	0.625		0.625	1.000
	0.583	1.000		0.625	1.000
	**Test4-Sensitivity**	P-value		**Test4-Specitivity**	P-value
	0.583	1.000		0.667	1.000
	0.625	1.000		0.625	1.000
	0.625	1.000		0.667	1.000
	0.625	0.375		0.625	1.000
	0.708	0.500		0.583	1.000
	**Test5-Sensitivity**	P-value		**Test5-Specitivity**	P-value
	0.542	1.000		0.708	1.000
	0.583	1.000		0.667	1.000
	0.625	1.000		0.667	1.000
	0.500	1.000		0.667	1.000
	0.625	1.000		0.667	1.000
	**Test6-Sensitivity**	P-value		**Test6-Specitivity**	P-value
0.583	0.583	1.000	0.708	0.708	1.000
0.583	0.500	0.625	0.625	0.792	0.125
0.625	0.417	0.063	0.667	0.833	0.125
0.500	0.458	1.000	0.667	0.792	0.250
0.625	0.500	0.250	0.625	0.792	0.125
	**Test7-Sensitivity**	P-value		**Test7-Specitivity**	P-value
	0.542	1.000		0.708	1.000
	0.583	1.000		0.667	1.000
	0.625	1.000		0.667	1.000
	0.583	0.500		0.667	1.000
	0.625	1.000		0.667	1.000
	**Test8-Sensitivity**	P-value		**Test8-Specitivity**	P-value
	0.583	1.000		0.708	1.000
	0.542	1.000		0.625	1.000
	0.625	1.000		0.667	1.000
	0.542	1.000		0.667	1.000
	0.625	1.000		0.667	1.000
	**Test9-Sensitivity**	P-value		**Test9-Specitivity**	P-value
	0.458	0.065		0.708	0.219
	0.583	0.344		0.625	0.219
	0.667	0.344		0.583	0.016*
	0.625	0.508		0.625	0.016*
	0.625	0.146		0.625	0.031*
	**Test10-Sensitivity**	P-value		**Test10-Specitivity**	P-value
	0.583	1.000		0.708	1.000
	0.625	1.000		0.625	1.000
	0.583	1.000		0.667	1.000
	0.583	0.625		0.625	1.000
	0.583	1.000		0.667	1.000

* $P < 0.05$

Abbreviations: AUC, area under curve; CI, Confidence interval

Supplementary Table 10. DeLong and McNemar Test Results for the Unstable Model across 10 Test Sets (Reference: Test2).

**Test2-AUC**	**Test1-AUC**	P-value	95%CI
	0.693	0.391	[-0.047 0.120]
	0.714	0.261	[-0.119 0.032]
	0.719	0.448	[-0.047 0.106]
	0.689	0.132	[-0.017 0.132]
	0.689	0.300	[-0.036 0.115]
	**Test3-AUC**	P-value	95%CI
	0.738	0.917	[-0.173 0.155]
	0.642	0.714	[-0.121 0.176]
	0.724	0.783	[-0.149 0.198]
	0.691	0.543	[-0.123 0.235]
	0.707	0.805	[-0.157 0.202]
	**Test4-AUC**	P-value	95%CI
	0.594	0.223	[-0.082 0.353]
	0.630	0.685	[-0.153 0.233]
	0.616	0.235	[-0.086 0.350]
	0.597	0.159	[-0.058 0.357]
	0.602	0.256	[-0.092 0.345]
	**Test5-AUC**	P-value	95%CI
	0.502	0.011*	[0.053 0.402]
	0.588	0.317	[-0.079 0.244]
	0.582	0.076	[-0.018 0.351]
	0.477	0.026*	[ ]
	0.578	0.088	[-0.023 0.325]
	**Test6-AUC**	P-value	95%CI
0.729	0.557	0.117	[-0.043 0.387]
0.670	0.554	0.316	[-0.111 0.344]
0.748	0.556	0.098	[-0.036 0.421]
0.747	0.540	0.057	[-0.006 0.419]
0.729	0.547	0.100	[-0.035 0.399]
	**Test7-AUC**	P-value	95%CI
	0.575	0.225	[ ]
	0.558	0.218	[-0.066 0.290]
	0.545	0.054	[-0.003 0.409]
	0.53	0.065	[ ]
	0.507	0.032*	[0.019 0.426]
	**Test8-AUC**	P-value	95%CI
	0.720	0.721	[-0.039 0.056]
	0.708	0.304	[-0.111 0.035]
	0.727	0.454	[-0.034 0.075]
	0.714	0.210	[-0.019 0.085]
	0.707	0.366	[-0.026 0.071]
	**Test9-AUC**	P-value	95%CI
	0.453	0.019*	[ ]
	0.521	0.080	[ ]
	0.659	0.419	[ ]
	0.491	0.029*	[ ]
	0.491	0.044*	[ ]
	**Test10-AUC**	P-value	95%CI
	0.804	0.354	[-0.232 0.083]
	0.795	0.084	[-0.267 0.017]
	0.793	0.470	[-0.168 0.077]
	0.804	0.418	[-0.196 0.081]
	0.806	0.249	[-0.206 0.054]

**Test2-Sensitivity**	**Test1-Sensitivity**	P-value	**Test2-Specitivity**	**Test1-Specitivity**	P-value
	0.875	1.000		0.458	1.000
	1.000	1.000		0.250	0.250
	1.000	1.000		0.375	0.500
	0.958	1.000		0.375	0.250
	0.917	1.000		0.292	1.000
	**Test3-Sensitivity**	P-value		**Test3-Specitivity**	P-value
	0.625	0.180		0.750	0.039*
	0.875	0.250		0.250	0.453
	0.792	0.063		0.458	0.125
	0.625	0.008*		0.625	0.004*
	0.833	0.688		0.417	0.125
	**Test4-Sensitivity**	P-value		**Test4-Specitivity**	P-value
	0.125	<0.001*		0.917	<0.001*
	0.833	0.012*		0.333	1.80E-01
	0.375	<0.001*		0.792	0.002*
	0.125	<0.001*		0.917	<0.001*
	0.375	<0.001*		0.667	0.013*
	**Test5-Sensitivity**	P-value		**Test5-Specitivity**	P-value
	0	<0.001*		1.000	<0.001*
	1.000	1.000		0.083	1.000
	0.583	0.002*		0.625	0.008*
	0	<0.001*		1.000	<0.001*
	0.042	<0.001*		0.958	<0.001*
	**Test6-Sensitivity**	P-value		**Test6-Specitivity**	P-value
0.833	0.042	<0.001*	0.458	1.000	<0.001*
1.000	0	<0.001*	0.125	1.000	<0.001*
1.000	0.042	<0.001*	0.292	1.000	<0.001*
0.958	0.042	<0.001*	0.25	1.000	<0.001*
0.917	0.042	<0.001*	0.25	1.000	<0.001*
	**Test7-Sensitivity**	P-value		**Test7-Specitivity**	P-value
	0	<0.001*		1.000	<0.001*
	0.958	1.000		0.042	0.625
	0.500	<0.001*		0.667	0.004*
	0	<0.001*		1.000	<0.001*
	0	<0.001*		1.000	<0.001*
	**Test8-Sensitivity**	P-value		**Test8-Specitivity**	P-value
	0.875	1.000		0.458	1.000
	1.000	1.000		0.167	1.000
	1.000	1.000		0.250	1.000
	0.958	1.000		0.250	1.000
	0.958	1.000		0.208	1.000
	**Test9-Sensitivity**	P-value		**Test9-Specitivity**	P-value
	0	<0.001*		1.000	<0.001*
	1.000	<0.001*		0.042	0.025*
	0.417	<0.001*		0.792	0.003*
	0	<0.001*		1.000	<0.001*
	0	<0.001*		1.000	<0.001*
	**Test10-Sensitivity**	P-value		**Test10-Specitivity**	P-value
	0.625	0.063		0.792	0.008*
	0.625	0.004*		0.750	<0.001*
	0.625	0.004*		0.792	<0.001*
	0.667	0.016*		0.792	<0.001*
	0.667	0.031*		0.792	<0.001*

* $P < 0.05$

Abbreviations: AUC, area under curve; CI, Confidence interval

Supplementary Table 11. DeLong and McNemar Test Results for the Intermediate stable Model across 10 Test Sets (Reference: Test2).

**Test2-AUC**	**Test1-AUC**	P-value	95%CI
	0.727	0.228	[-0.050 0.012]
	0.575	0.158	[-0.018 0.108]
	0.745	0.088	[-0.067 0.005]
	0.701	0.120	[-0.122 0.014]
	0.767	0.457	[-0.023 0.050]
	**Test3-AUC**	P-value	95%CI
	0.655	0.432	[-0.081 0.188]
	0.545	0.622	[ ]
	0.665	0.432	[-0.073 0.170]
	0.616	0.692	[-0.123 0.186]
	0.720	0.480	[-0.108 0.229]
	**Test4-AUC**	P-value	95%CI
	0.602	0.263	[-0.079 0.291]
	0.593	0.851	[ ]
	0.620	0.211	[-0.053 0.241]
	0.469	0.171	[ ]
	0.608	0.109	[-0.039 0.386]
	**Test5-AUC**	P-value	95%CI
	0.660	0.203	[-0.026 0.123]
	0.436	0.027*	[0.021 0.347]
	0.694	0.518	[-0.039 0.077]
	0.620	0.513	[-0.055 0.111]
	0.759	0.453	[-0.036 0.082]
	**Test6-AUC**	P-value	95%CI
0.708	0.714	0.418	[-0.166 0.069]
0.620	0.630	0.201	[-0.352 0.074]
0.714	0.745	0.672	[-0.078 0.050]
0.648	0.651	0.722	[-0.226 0.157]
0.781	0.776	0.849	[-0.078 0.064]
	**Test7-AUC**	P-value	95%CI
	0.618	0.118	[-0.023 0.203]
	0.499	0.149	[-0.043 0.285]
	0.670	0.360	[-0.050 0.136]
	0.609	0.456	[-0.062 0.139]
	0.743	0.316	[-0.036 0.113]
	**Test8-AUC**	P-value	95%CI
	0.757	0.676	[-0.030 0.019]
	0.759	0.623	[-0.052 0.031]
	0.727	0.121	[-0.071 0.008]
	0.682	0.812	[-0.032 0.025]
	0.788	0.480	[-0.009 0.020]
	**Test9-AUC**	P-value	95%CI
	0.552	0.173	[ ]
	0.500	0.158	[ ]
	0.542	0.145	[ ]
	0.569	0.511	[ ]
	0.665	0.276	[ ]
	**Test10-AUC**	P-value	95%CI
	0.767	0.372	[-0.189 0.071]
	0.767	0.186	[-0.366 0.071]
	0.743	0.457	[-0.107 0.048]
	0.771	0.076	[-0.259 0.013]
	0.788	0.897	[-0.112 0.098]

**Test2-Sensitivity**	**Test1-Sensitivity**	P-value	**Test2-Specitivity**	**Test1-Specitivity**	P-value
	0.083	1.000		1.000	1.000
	0.042	1.000		1.000	1.000
	0.208	1.000		0.917	1.000
	0.083	1.000		1.000	1.000
	0.042	1.000		1.000	1.000
	**Test3-Sensitivity**	P-value		**Test3-Specitivity**	P-value
	0.042	1.000		1.000	1.000
	0	0.500		1.000	1.000
	0.083	0.500		0.958	1.000
	0.042	1.000		0.958	1.000
	0.042	1.000		1.000	1.000
	**Test4-Sensitivity**	P-value		**Test4-Specitivity**	P-value
	0.083	1.000		1.000	1.000
	0	0.500		1.000	1.000
	0.125	1.000		0.917	1.000
	0.125	1.000		0.958	1.000
	0.083	1.000		1.000	1.000
	**Test5-Sensitivity**	P-value		**Test5-Specitivity**	P-value
	0	0.500		1.000	1.000
	0	0.500		1.000	1.000
	0	0.125		1.000	0.500
	0	0.500		1.000	1.000
	0	1.000		1.000	1.000
	**Test6-Sensitivity**	P-value		**Test6-Specitivity**	P-value
0.083	0.042	1.000	1.000	1.000	1.000
0.083	0.042	1.000	1.000	0.958	1.000
0.167	0.125	1.000	0.917	0.917	1.000
0.083	0.042	1.000	1.000	1.000	1.000
0.042	0	1.000	1.000	1.000	1.000
	**Test7-Sensitivity**	P-value		**Test7-Specitivity**	P-value
	0	0.500		1.000	1.000
	0	0.500		1.000	1.000
	0	0.125		1.000	0.500
	0	0.500		1.000	1.000
	0	1.000		1.000	1.000
	**Test8-Sensitivity**	P-value		**Test8-Specitivity**	P-value
	0.083	1.000		1.000	1.000
	0.042	1.000		1.000	1.000
	0.167	1.000		0.917	1.000
	0.042	1.000		1.000	1.000
	0.042	1.000		1.000	1.000
	**Test9-Sensitivity**	P-value		**Test9-Specitivity**	P-value
	0	0.500		1.000	1.000
	0	0.500		1.000	1.000
	0	0.125		1.000	0.500
	0	0.500		1.000	1.000
	0	1.000		1.000	1.000
	**Test10-Sensitivity**	P-value		**Test10-Specitivity**	P-value
	0.542	0.001*		0.792	0.063
	0.458	0.004*		0.833	0.125
	0.500	0.008*		0.792	0.250
	0.542	0.001*		0.833	0.125
	0.333	0.016*		0.875	0.250

* $P < 0.05$

Abbreviations: AUC, area under curve; CI, Confidence interval

Section 2. Additional Experiments

Ablation Study of Feature Combination Models

To further investigate the effect of features with different stability, we constructed three additional models combining features of different stability levels:

- **SI model: Stable + Intermediate stable features**
- **UI model: Unstable + Intermediate stable features**
- **SU model: Stable + Unstable features**

The SI and UI models were compared with the Intermediate stable model, while the SU model was compared with the Unstable model. Performance comparisons were conducted on the training set, validation set, and Test Set 2 using DeLong and McNemar tests. Furthermore, to assess the models' generalizability across varying CT imaging conditions, the trained models from each fold were applied to Test Sets 1–10, and their performance was compared with Test Set 2 as the reference.

Summary of Findings: The radiomic features selected for the final SI and UI models were predominantly intermediate stable features, resulting in performance largely consistent with that of the Intermediate stable model.

In contrast, the SU model incorporated a nearly equal proportion of stable and unstable features. While it demonstrated greater robustness than the Unstable model across the ten CT-specific test sets, its performance was still less stable compared with the Stable model.

See **Supplementary Table 12-16**, **Supplementary Figure2** for details.

Supplementary Table 12. Five-fold average performance metrics of the additional three models across each dataset.

	SI model	SU model	UI model
**Train set**			
AUC	0.871	0.854	0.871
Accuracy	0.785	0.780	0.783
Sensitivity	0.785	0.781	0.793
Specificity	0.786	0.780	0.774
G-mean	0.785	0.780	0.783
F1-score	0.785	0.780	0.785
**Valid set**			
AUC	0.859	0.838	0.857
Accuracy	0.776	0.762	0.769
Sensitivity	0.769	0.765	0.770
Specificity	0.783	0.760	0.769
G-mean	0.776	0.762	0.770
F1-score	0.775	0.763	0.770
**Test set1**			
AUC	0.748	0.730	0.741
Accuracy	0.550	0.663	0.538
Sensitivity	0.150	0.600	0.117
Specificity	0.950	0.725	0.958
G-mean	0.345	0.658	0.319
F1-score	0.228	0.639	0.193
**Test set2**			
AUC	0.732	0.705	0.730
Accuracy	0.554	0.65	0.533
Sensitivity	0.158	0.600	0.108
Specificity	0.950	0.700	0.958
G-mean	0.351	0.647	0.310
F1-score	0.235	0.631	0.182
**Test set3**			
AUC	0.697	0.702	0.661
Accuracy	0.529	0.633	0.517
Sensitivity	0.067	0.583	0.067
Specificity	0.992	0.684	0.967
G-mean	0.254	0.630	0.250
F1-score	0.123	0.613	0.120
**Test set4**			
AUC	0.663	0.610	0.594
Accuracy	0.534	0.546	0.529
Sensitivity	0.075	0.433	0.083
Specificity	0.992	0.658	0.975
G-mean	0.266	0.532	0.277
F1-score	0.136	0.485	0.147
**Test set5**			
AUC	0.718	0.610	0.713
Accuracy	0.508	0.608	0.504
Sensitivity	0.025	0.300	0.017
Specificity	0.992	0.917	0.992

G-mean	0.069	0.447	0.056
F1-score	NA	NA	NA
**Test set6**			
AUC	0.752	0.659	0.733
Accuracy	0.533	0.500	0.513
Sensitivity	0.109	0	0.034
Specificity	0.958	1.000	0.992
G-mean	0.238	0	0.163
F1-score	0.188	NA	NA
**Test set7**			
AUC	0.690	0.619	0.709
Accuracy	0.504	0.583	0.504
Sensitivity	0.017	0.225	0.017
Specificity	0.992	0.942	0.992
G-mean	0.056	0.383	0.056
F1-score	NA	NA	NA
**Test set8**			
AUC	0.740	0.705	0.733
Accuracy	0.554	0.654	0.533
Sensitivity	0.158	0.600	0.108
Specificity	0.950	0.708	0.958
G-mean	0.351	0.650	0.310
F1-score	0.235	0.633	0.182
**Test set9**			
AUC	0.651	0.604	0.694
Accuracy	0.500	0.504	0.500
Sensitivity	0.017	0.008	0.017
Specificity	0.983	1.000	0.983
G-mean	0.055	0.041	0.055
F1-score	NA	NA	NA
**Test set10**			
AUC	0.773	0.757	0.759
Accuracy	0.650	0.675	0.621
Sensitivity	0.492	0.550	0.433
Specificity	0.809	0.800	0.808
G-mean	0.626	0.663	0.585
F1-score	0.580	0.628	0.526

Abbreviations: AUC, area under curve

Supplementary Table 13. Fold-wise DeLong and McNemar Test Results for Pairwise Comparisons among three additional Models on the Training Set, Valid Set and Test Set2.

**Training Set**			
Fold	Intermediate-AUC	SI-AUC	P-value
1	0.877	0.877	1.000
2	0.880	0.878	0.207
3	0.868	0.867	0.678
4	0.862	0.859	0.354
5	0.877	0.875	0.655
Fold	Intermediate-AUC	UI-AUC	P-value
1	0.877	0.870	0.010*
2	0.880	0.878	0.159
3	0.868	0.865	0.419
4	0.862	0.865	0.182
5	0.877	0.875	0.385
Fold	Unstable-AUC	SU-AUC	P-value
1	0.822	0.853	＜0.001*
2	0.836	0.859	0.003*
3	0.826	0.855	＜0.001*
4	0.814	0.843	＜0.001*
5	0.839	0.858	0.001*
**Valid Set**			
Fold	Intermediate-AUC	SI-AUC	P-value
1	0.854	0.854	0.109
2	0.826	0.833	0.243
3	0.878	0.879	0.916
4	0.892	0.897	0.455
5	0.831	0.83	0.947
Fold	Intermediate-AUC	UI-AUC	P-value
1	0.854	0.856	0.673
2	0.826	0.834	0.137
3	0.878	0.869	0.205
4	0.892	0.892	0.930
5	0.831	0.833	0.622
Fold	Unstable-AUC	SU-AUC	P-value
1	0.805	0.847	0.033*
2	0.765	0.815	0.025*
3	0.822	0.844	0.118
4	0.871	0.873	0.862
5	0.777	0.809	0.049*
**Test Set2**			
Fold	Intermediate-AUC	SI-AUC	P-value
1	0.708	0.707	0.480
2	0.620	0.705	0.403
3	0.714	0.762	0.096
4	0.648	0.703	0.325
5	0.781	0.783	0.953
Fold	Intermediate-AUC	UI-AUC	P-value
1	0.708	0.717	0.728
2	0.620	0.719	0.312
3	0.714	0.734	0.506
4	0.648	0.689	0.354
5	0.781	0.793	0.538
Fold	Unstable-AUC	SU-AUC	P-value
1	0.729	0.655	0.264
2	0.670	0.745	0.316
3	0.748	0.644	0.270
4	0.747	0.734	0.856
5	0.729	0.748	0.763

**Training Set**						
Fold	Intermediate-Sensitivity	SI-Sensitivity	P-value	Intermediate-Specificity	SI-Specificity	P-value
1	0.791	0.791	1.000	0.793	0.793	1.000
2	0.795	0.793	1.000	0.776	0.785	0.359
3	0.771	0.780	0.473	0.791	0.789	0.090
4	0.791	0.779	0.557	0.775	0.782	0.011*
5	0.793	0.780	1.000	0.775	0.782	0.099
Fold	Intermediate-Sensitivity	UI-Sensitivity	P-value	Intermediate-Specificity	UI-Specificity	P-value
1	0.791	0.791	1.000	0.793	0.775	0.041*
2	0.795	0.798	1.000	0.776	0.773	0.607
3	0.771	0.791	0.143	0.791	0.769	0.079
4	0.791	0.782	1.000	0.775	0.780	0.296
5	0.793	0.802	0.690	0.775	0.773	0.648
Fold	Unstable-Sensitivity	SU-Sensitivity	P-value	Unstable-Specificity	SU-Specificity	P-value
1	0.824	0.782	0.014*	0.673	0.779	＜0.001*
2	0.813	0.791	＜0.001*	0.676	0.773	0.042*
3	0.798	0.775	＜0.001*	0.665	0.785	0.002*
4	0.802	0.760	＜0.001*	0.660	0.773	＜0.001*
5	0.818	0.796	0.001*	0.704	0.789	0.005*
**Valid Set**						
Fold	Intermediate-Sensitivity	SI-Sensitivity	P-value	Intermediate-Specificity	SI-Specificity	P-value
1	0.752	0.752	1.000	0.766	0.766	1.000
2	0.754	0.768	1.000	0.761	0.783	1.000
3	0.775	0.768	0.219	0.826	0.819	0.375
4	0.825	0.803	0.227	0.797	0.826	0.688
5	0.739	0.754	0.453	0.752	0.723	0.453
Fold	Intermediate-Sensitivity	UI-Sensitivity	P-value	Intermediate-Specificity	UI-Specificity	P-value
1	0.752	0.766	0.625	0.766	0.752	0.625
2	0.754	0.754	1.000	0.761	0.761	1.000
3	0.775	0.761	0.508	0.826	0.790	1.000
4	0.825	0.825	0.549	0.797	0.812	1.000
5	0.739	0.746	0.727	0.752	0.730	0.500
Fold	Unstable-Sensitivity	SU-Sensitivity	P-value	Unstable-Specificity	SU-Specificity	P-value
1	0.774	0.745	0.541	0.693	0.781	0.043*
2	0.768	0.790	0.345	0.638	0.710	0.029*
3	0.783	0.732	0.189	0.725	0.812	0.023*
4	0.869	0.825	0.167	0.739	0.768	0.648
5	0.790	0.732	0.002*	0.606	0.730	0.096
**Test Set2**						
Fold	Intermediate-Sensitivity	SI-Sensitivity	P-value	Intermediate-Specificity	SI-Specificity	P-value
1	0.083	0.083	1.000	1.000	1.000	1.000
2	0.083	0.417	0.008	1.000	0.792	0.063
3	0.167	0.167	1.000	0.917	0.958	1.000
4	0.083	0.083	1.000	1.000	1.000	1.000
5	0.042	0.042	1.000	1.000	1.000	1.000
Fold	Intermediate-Sensitivity	UI-Sensitivity	P-value	Intermediate-Specificity	UI-Specificity	P-value
1	0.083	0.083	1.000	1.000	1.000	1.000
2	0.083	0.125	1.000	1.000	0.917	0.500
3	0.167	0.208	1.000	0.917	0.917	1.000
4	0.083	0.083	1.000	1.000	0.958	1.000
5	0.042	0.042	1.000	1.000	1.000	1.000
Fold	Unstable-Sensitivity	SU-Sensitivity	P-value	Unstable-Specificity	SU-Specificity	P-value
1	0.833	0.542	0.016*	0.458	0.750	0.016*
2	1.000	0.625	0.004*	0.125	0.667	＜0.001*
3	1.000	0.542	0.001*	0.292	0.750	0.001*
4	0.958	0.625	0.021*	0.250	0.667	0.002*
5	0.917	0.667	0.070	0.250	0.667	0.002*

* $P < 0.05$

Abbreviations: AUC, area under curve; CI, Confidence interval

Supplementary Table 14. DeLong and McNemar Test Results for the SI Model across 10 Test Sets (Reference: Test2).

**Test2-AUC**	**Test1-AUC**	P-value	95%CI
	0.729	0.144	[-0.053 0.008]
	0.707	0.908	[-0.031 0.028]
	0.776	0.365	[-0.044 0.016]
	0.745	0.080	[-0.088 0.005]
	0.785	0.890	[-0.026 0.023]
	**Test3-AUC**	P-value	95%CI
	0.651	0.414	[-0.078 0.189]
	0.653	0.317	[-0.050 0.154]
	0.757	0.711	[-0.086 0.096]
	0.689	0.787	[-0.087 0.114]
	0.733	0.529	[-0.106 0.207]
	**Test4-AUC**	P-value	95%CI
	0.594	0.240	[-0.075 0.301]
	0.682	0.742	[-0.112 0.157]
	0.710	0.365	[-0.061 0.165]
	0.691	0.862	[-0.125 0.149]
	0.637	0.147	[-0.051 0.343]
	**Test5-AUC**	P-value	95%CI
	0.661	0.237	[-0.030 0.120]
	0.712	0.750	[-0.050 0.036]
	0.757	0.847	[-0.048 0.058]
	0.698	0.885	[-0.065 0.076]
	0.764	0.596	[-0.051 0.090]
	**Test6-AUC**	P-value	95%CI
0.707	0.760	0.360	[-0.169 0.061]
0.705	0.731	0.486	[-0.099 0.047]
0.762	0.771	0.864	[-0.108 0.091]
0.703	0.710	0.939	[-0.184 0.170]
0.783	0.786	0.942	[-0.098 0.091]
	**Test7-AUC**	P-value	95%CI
	0.618	0.125	[-0.024 0.202]
	0.710	0.868	[-0.067 0.056]
	0.747	0.674	[-0.057 0.088]
	0.681	0.630	[-0.069 0.114]
	0.696	0.221	[-0.052 0.226]
	**Test8-AUC**	P-value	95%CI
	0.715	0.488	[-0.033 0.016]
	0.710	0.524	[-0.021 0.011]
	0.773	0.452	[-0.038 0.017]
	0.714	0.519	[-0.042 0.021]
	0.790	0.529	[-0.029 0.015]
	**Test9-AUC**	P-value	95%CI
	0.550	0.163	[ ]
	0.719	0.898	[ ]
	0.743	0.851	[ ]
	0.684	0.863	[ ]
	0.561	0.044	[ ]
	**Test10-AUC**	P-value	95%CI
	0.769	0.345	[-0.192 0.067]
	0.771	0.132	[-0.152 0.020]
	0.786	0.543	[-0.103 0.054]
	0.755	0.257	[-0.142 0.038]
	0.785	0.963	[-0.074 0.071]

Test2-Sensitivity	Test1-Sensitivity	P-value	Test2-Specitivity	Test1-Specitivity	P-value
	0.083	1.000		1.000	1.000
	0.375	1.000		0.792	1.000
	0.167	1.000		0.958	1.000
	0.083	1.000		1.000	1.000
	0.042	1.000		1.000	1.000
	**Test3-Sensitivity**	P-value		**Test3-Specitivity**	P-value
	0.042	1.000		1.000	1.000
	0.083	0.008*		0.958	0.125
	0.083	0.500		1.000	1.000
	0.083	1.000		1.000	1.000
	0.042	1.000		1.000	1.000
	**Test4-Sensitivity**	P-value		**Test4-Specitivity**	P-value
	0.083	1.000		1.000	1.000
	0.125	0.039*		0.958	0.125
	0.042	0.250		1.000	1.000
	0.083	1.000		1.000	1.000
	0.042	1.000		1.000	1.000
	**Test5-Sensitivity**	P-value		**Test5-Specitivity**	P-value
	0	0.500		1.000	1.000
	0.125	0.016*		0.958	0.125
	0	0.125		1.000	1.000
	0	0.500		1.000	1.000
	0	1.000		1.000	1.000
	**Test6-Sensitivity**	P-value		**Test6-Specitivity**	P-value
0.083	0.042	1.000	1.000	1.000	1.000
0.417	0.417	1.000	0.792	0.792	1.000
0.167	0.042	0.250	0.958	1.000	1.000
0.083	0.042	1.000	1.000	1.000	1.000
0.042	0	1.000	1.000	1.000	1.000
	**Test7-Sensitivity**	P-value		**Test7-Specitivity**	P-value
	0	0.500		1.000	1.000
	0.083	0.008*		0.958	0.125
	0	0.125		1.000	1.000
	0	0.500		1.000	1.000
	0	1.000		1.000	1.000
	**Test8-Sensitivity**	P-value		**Test8-Specitivity**	P-value
	0.083	1.000		1.000	1.000
	0.417	1.000		0.792	1.000
	0.167	1.000		0.958	1.000
	0.083	1.000		1.000	1.000
	0.042	1.000		1.000	1.000
	**Test9-Sensitivity**	P-value		**Test9-Specitivity**	P-value
	0	0.500		1.000	1.000
	0.083	0.021*		0.917	0.375
	0	0.125		1.000	1.000
	0	0.500		1.000	1.000
	0	1.000		1.000	1.000
	**Test10-Sensitivity**	P-value		**Test10-Specitivity**	P-value
	0.542	0.001*		0.792	0.063
	0.500	0.688		0.792	1.000
	0.542	0.004*		0.792	0.125
	0.542	0.001*		0.750	0.031*
	0.333	0.016*		0.917	0.500

* $P < 0.05$

Abbreviations: AUC, area under curve; CI, Confidence interval

Supplementary Table 15. DeLong and McNemar Test Results for the SU Model across 10 Test Sets (Reference: Test2).

**Test2-AUC**	**Test1-AUC**	P-value	95%CI
	0.693	0.139	[-0.089 0.012]
	0.767	0.268	[-0.062 0.017]
	0.674	0.264	[-0.081 0.022]
	0.759	0.322	[-0.072 0.024]
	0.755	0.789	[-0.058 0.044]
	**Test3-AUC**	P-value	95%CI
	0.675	0.739	[-0.143 0.102]
	0.734	0.836	[-0.088 0.109]
	0.649	0.941	[-0.142 0.132]
	0.727	0.887	[-0.089 0.103]
	0.727	0.712	[-0.090 0.132]
	**Test4-AUC**	P-value	95%CI
	0.58	0.459	[-0.123 0.272]
	0.622	0.100	[-0.024 0.270]
	0.589	0.618	[-0.163 0.274]
	0.632	0.194	[-0.052 0.257]
	0.625	0.157	[-0.048 0.294]
	**Test5-AUC**	P-value	95%CI
	0.469	0.179	[ ]
	0.710	0.354	[-0.039 0.108]
	0.481	0.268	[ ]
	0.691	0.248	[-0.030 0.117]
	0.701	0.220	[-0.028 0.122]
	**Test6-AUC**	P-value	95%CI
0.655	0.663	0.941	[-0.240 0.222]
0.745	0.681	0.582	[-0.165 0.293]
0.644	0.628	0.896	[-0.218 0.249]
0.734	0.674	0.608	[-0.171 0.293]
0.748	0.651	0.413	[-0.135 0.330]
	**Test7-AUC**	P-value	95%CI
	0.505	0.276	[ ]
	0.701	0.317	[-0.042 0.128]
	0.493	0.311	[ ]
	0.686	0.259	[-0.036 0.133]
	0.708	0.434	[-0.060 0.140]
	**Test8-AUC**	P-value	95%CI
	0.675	0.226	[-0.055 0.013]
	0.740	0.524	[-0.011 0.021]
	0.653	0.606	[-0.042 0.024]
	0.727	0.493	[-0.013 0.027]
	0.731	0.189	[-0.009 0.043]
	**Test9-AUC**	P-value	95%CI
	0.635	0.861	[ ]
	0.613	0.237	[ ]
	0.632	0.916	[ ]
	0.519	0.060	[ ]
	0.620	0.242	[ ]
	**Test10-AUC**	P-value	95%CI
	0.752	0.162	[-0.233 0.039]
	0.762	0.655	[-0.093 0.059]
	0.790	0.071	[-0.304 0.013]
	0.722	0.766	[-0.068 0.092]
	0.760	0.762	[-0.091 0.066]

Test2-Sensitivity	Test1-Sensitivity	P-value	Test2-Specitivity	Test1-Specitivity	P-value
	0.542	1.000		0.792	1.000
	0.625	1.000		0.708	1.000
	0.542	1.000		0.750	1.000
	0.625	1.000		0.708	1.000
	0.667	1.000		0.667	1.000
	**Test3-Sensitivity**	P-value		**Test3-Specitivity**	P-value
	0.542	1.000		0.750	1.000
	0.625	1.000		0.667	1.000
	0.500	1.000		0.667	0.688
	0.625	1.000		0.667	1.000
	0.625	1.000		0.667	1.000
	**Test4-Sensitivity**	P-value		**Test4-Specitivity**	P-value
	0.333	0.180		0.625	0.453
	0.500	0.453		0.708	1.000
	0.333	0.180		0.667	0.727
	0.500	0.453		0.667	1.000
	0.500	0.289		0.625	1.000
	**Test5-Sensitivity**	P-value		**Test5-Specitivity**	P-value
	0	<0.001*		1.000	0.031*
	0.458	0.125		0.875	0.063
	0.125	0.002*		1.000	0.031*
	0.500	0.250		0.833	0.125
	0.417	0.031*		0.875	0.063
	**Test6-Sensitivity**	P-value		**Test6-Specitivity**	P-value
0.542	0	<0.001*	0.750	1.000	0.031*
0.625	0	<0.001*	0.667	1.000	0.008*
0.542	0	<0.001*	0.750	1.000	0.031*
0.625	0	<0.001*	0.667	1.000	0.008*
0.667	0	<0.001*	0.667	1.000	0.008*
	**Test7-Sensitivity**	P-value		**Test7-Specitivity**	P-value
	0	<0.001*		1.000	0.031*
	0.333	0.016*		0.917	0.031*
	0.042	<0.001*		1.000	0.031*
	0.375	0.031*		0.875	0.063
	0.375	0.016*		0.917	0.031*
	**Test8-Sensitivity**	P-value		**Test8-Specitivity**	P-value
	0.542	1.000		0.792	1.000
	0.625	1.000		0.708	1.000
	0.542	1.000		0.750	1.000
	0.625	1.000		0.667	1.000
	0.667	1.000		0.625	1.000
	**Test9-Sensitivity**	P-value		**Test9-Specitivity**	P-value
	0	<0.001*		1.000	0.031*
	0	<0.001*		1.000	0.008*
	0.042	<0.001*		1.000	0.125
	0	<0.001*		1.000	0.008*
	0	<0.001*		1.000	0.008*
	**Test10-Sensitivity**	P-value		**Test10-Specitivity**	P-value
	0.542	1.000		0.792	1.000
	0.583	1.000		0.750	0.500
	0.542	1.000		0.792	1.000
	0.542	0.500		0.833	0.125
	0.542	0.250		0.833	0.125

* $P < 0.05$

Abbreviations: AUC, area under curve; CI, Confidence interval

Supplementary Table 16. DeLong and McNemar Test Results for the UI Model across 10 Test Sets (Reference: Test2).

**Test2-AUC**	**Test1-AUC**	P-value	95%CI
	0.738	0.254	[-0.057 0.015]
	0.729	0.480	[-0.039 0.018]
	0.750	0.442	[-0.055 0.024]
	0.705	0.533	[-0.065 0.033]
	0.785	0.647	[-0.028 0.046]
	**Test3-AUC**	P-value	95%CI
	0.691	0.667	[-0.093 0.145]
	0.653	0.262	[-0.049 0.181]
	0.616	0.063	[-0.006 0.242]
	0.613	0.207	[-0.042 0.195]
	0.734	0.436	[-0.090 0.208]
	**Test4-AUC**	P-value	95%CI
	0.628	0.292	[-0.076 0.253]
	0.656	0.353	[-0.069 0.194]
	0.550	0.031*	[0.017 0.351]
	0.533	0.072	[-0.014 0.326]
	0.601	0.059	[-0.007 0.393]
	**Test5-AUC**	P-value	95%CI
	0.691	0.462	[-0.043 0.095]
	0.696	0.442	[-0.035 0.080]
	0.710	0.466	[-0.041 0.090]
	0.688	0.965	[-0.077 0.080]
	0.778	0.554	[-0.036 0.067]
	**Test6-AUC**	P-value	95%CI
0.717	0.748	0.685	[-0.182 0.120]
0.719	0.741	0.728	[-0.150 0.104]
0.734	0.665	0.525	[-0.145 0.283]
0.689	0.738	0.563	[-0.213 0.116]
0.793	0.774	0.671	[-0.069 0.107]
	**Test7-AUC**	P-value	95%CI
	0.693	0.547	[-0.055 0.103]
	0.700	0.591	[-0.051 0.089]
	0.701	0.403	[-0.044 0.110]
	0.668	0.651	[-0.070 0.111]
	0.781	0.718	[-0.054 0.078]
	**Test8-AUC**	P-value	95%CI
	0.729	0.443	[-0.043 0.019]
	0.736	0.317	[-0.051 0.017]
	0.731	0.862	[-0.036 0.043]
	0.677	0.493	[-0.023 0.047]
	0.793	1.000	[-0.015 0.015]
	**Test9-AUC**	P-value	95%CI
	0.689	0.800	[ ]
	0.715	0.974	[ ]
	0.665	0.526	[ ]
	0.663	0.817	[ ]
	0.736	0.567	[ ]
	**Test10-AUC**	P-value	95%CI
	0.731	0.828	[-0.139 0.111]
	0.774	0.199	[-0.140 0.029]
	0.745	0.824	[-0.102 0.081]
	0.724	0.543	[-0.147 0.077]
	0.819	0.528	[-0.107 0.055]

Test2-Sensitivity	Test1-Sensitivity	P-value	Test2-Specitivity	Test1-Specitivity	P-value
	0.083	1.000		1.000	1.000
	0.083	1.000		0.917	1.000
	0.250	1.000		0.917	1.000
	0.125	1.000		0.958	1.000
	0.042	1.000		1.000	1.000
	**Test3-Sensitivity**	P-value		**Test3-Specitivity**	P-value
	0.042	1.000		1.000	1.000
	0.083	1.000		0.958	1.000
	0.083	0.250		0.917	1.000
	0.083	1.000		0.958	1.000
	0.042	1.000		1.000	1.000
	**Test4-Sensitivity**	P-value		**Test4-Specitivity**	P-value
	0.042	1.000		1.000	1.000
	0.083	1.000		0.958	1.000
	0.125	0.625		0.917	1.000
	0.125	1.000		1.000	1.000
	0.042	1.000		1.000	1.000
	**Test5-Sensitivity**	P-value		**Test5-Specitivity**	P-value
	0	0.500		1.000	1.000
	0.083	1.000		0.958	1.000
	0	0.063		1.000	0.500
	0	0.500		1.000	1.000
	0	1.000		1.000	1.000
	**Test6-Sensitivity**	P-value		**Test6-Specitivity**	P-value
0.083	0.042	1.000	1.000	1.000	1.000
0.125	0.042	0.500	0.917	0.958	1.000
0.208	0.042	0.125	0.917	1.000	0.500
0.083	0.042	1.000	0.958	1.000	1.000
0.042	0	1.000	1.000	1.000	1.000
	**Test7-Sensitivity**	P-value		**Test7-Specitivity**	P-value
	0	0.500		1.000	1.000
	0.083	1.000		0.958	1.000
	0	0.063		1.000	0.500
	0	0.500		1.000	1.000
	0	1.000		1.000	1.000
	**Test8-Sensitivity**	P-value		**Test8-Specitivity**	P-value
	0.083	1.000		1.000	1.000
	0.125	1.000		0.917	1.000
	0.208	1.000		0.917	1.000
	0.083	1.000		0.958	1.000
	0.042	1.000		1.000	1.000
	**Test9-Sensitivity**	P-value		**Test9-Specitivity**	P-value
	0	0.500		1.000	1.000
	0.083	1.000		0.917	1.000
	0	0.063		1.000	0.500
	0	0.500		1.000	1.000
	0	1.000		1.000	1.000
	**Test10-Sensitivity**	P-value		**Test10-Specitivity**	P-value
	0.542	0.001*		0.792	0.063
	0.458	0.008*		0.833	0.500
	0.500	0.016*		0.750	0.125
	0.417	0.008*		0.750	0.063
	0.250	0.063		0.917	0.500

* $P < 0.05$

Abbreviations: AUC, area under curve; CI, Confidence interval

Supplementary Figures

Supplementary Figure 1.

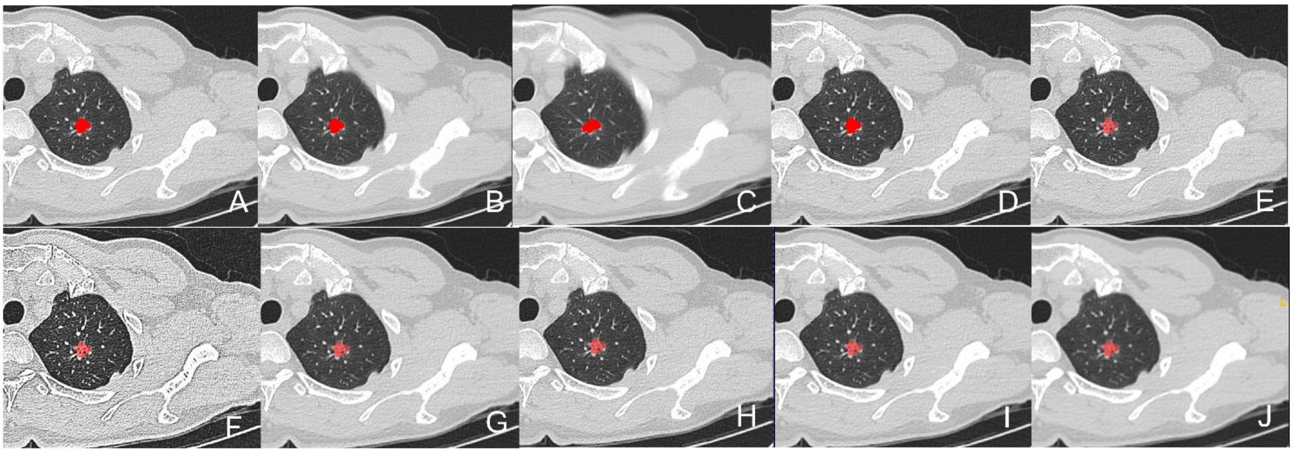

Segmentation results of images reconstructed using different CT parameters are presented. Except for F, which was exported from PACS, all others were exported directly from the workstation.A:1mm,B_SHARP_C,1024×1024;B:3mm,B_SHARP_C,1024×1024;C:5m,B_SHARP_C,1024×1024;D:1mm,B_SHARP_C,512×512;E:1mm,B_SHARP_C,1024×1024(PACS);F:1mm,B_VSHARP_D,1024×1024;G:1mm,B_SHARP_A,1024×1024;H:1mm,B_SOFT_F,1024×1024;I:1mm,B_SOFT_C,1024×1024;J:1mm,B_VSOFT_A,1024×1024.

Supplementary Figure 2.

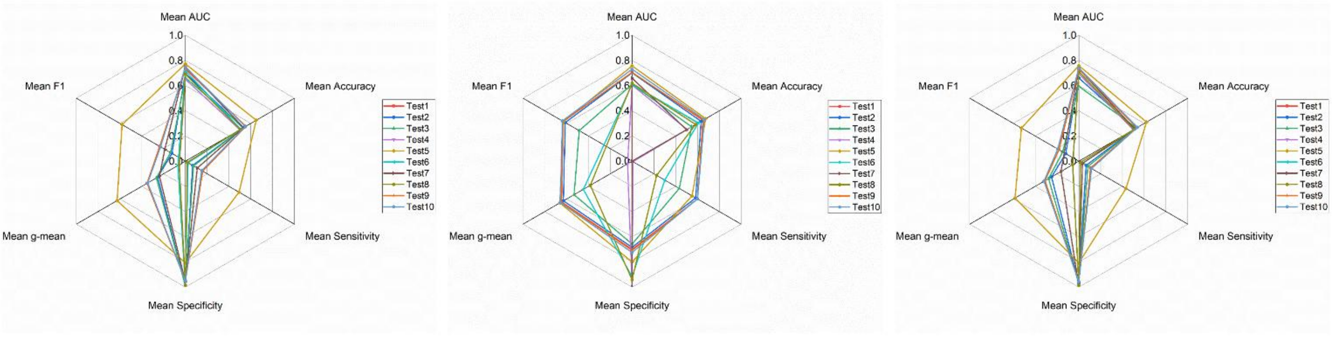

A B C

Radar plots showing the performance metrics of the SI model (A), SU model (B), and UI model (C) across ten test sets (based on the mean of five-fold cross-validation).
